# Actl6a regulates autophagy via Sox2-dependent Atg5 and Atg7 expression to inhibit apoptosis in spinal cord injury

**DOI:** 10.1016/j.jare.2025.01.038

**Published:** 2025-01-26

**Authors:** Jian Hao, Yubiao Yang, Li Xie, Zhenhan Li, Boyuan Ma, Bitao Wang, Jinyu Chen, Zhi Zeng, Xianhu Zhou

**Affiliations:** a Orthopedics Department, the Second Affliated Hospital, Guangzhou medical University, Guangzhou 510260, China; b Key Laboratory for Stem Cells and Tissue Engineering (Sun Yat-sen University), Ministry of Education, Guangzhou, 510080, China; c Institute of Spinal Cord Injury, Sun Yat-sen University, Guangzhou, 510120, China; d Department of Anesthesiology, Qilu Hospital of Shandong University Dezhou Hospital, Dezhou, China

**Keywords:** Spinal cord injury, Autophagy, Neuron, Sox2, Atg5, Atg7

## Abstract

**Introduction:**

Spinal cord injury (SCI) is a severe central nervous system disorder with limited treatment options. While autophagy plays a protective role in neural repair, its regulatory mechanisms in SCI remain unclear. Actin-like protein 6A (Actl6a) influences cell fate and neural development, yet its specific role in SCI repair is not well understood. This study investigates Actl6a’s function in regulating autophagy and apoptosis via the transcription factor Sox2 in SCI.

**Objectives:**

This study aims to determine if Actl6a promotes neural survival post-SCI by regulating autophagy-related genes Atg5 and Atg7 through Sox2. It also examines how the demethylase Fto modulates Actl6a mRNA stability via m6A methylation.

**Methods:**

In vitro experiments were conducted using primary neurons and HT-22 hippocampal cells exposed to hydrogen peroxide (H_2_O_2_)-induced oxidative stress. Actl6a expression was manipulated by knockdown or overexpression. For in vivo studies, a rat SCI model was established with AAV-Actl6a injected at the injury site to induce Actl6a overexpression. Autophagy and apoptosis markers were analyzed using immunofluorescence, Western blotting, and qPCR. Additionally, m6A dot blot and RNA immunoprecipitation (RIP) assays were performed to assess Fto’s role in regulating Actl6a mRNA methylation and stability.

**Results:**

Actl6a expression significantly decreased after SCI, resulting in increased apoptosis. Overexpressing Actl6a enhanced autophagy, reduced apoptosis, and improved neurological function in SCI models. Mechanistically, Actl6a and Sox2 collaboratively upregulated Atg5 and Atg7 expression, promoting autophagy. Fto’s modulation of Actl6a mRNA stability via m6A demethylation further influenced autophagy and apoptosis.

**Conclusion:**

Actl6a, through interaction with Sox2, plays a critical role in modulating autophagy and reducing apoptosis in SCI, with Fto’s m6A modification affecting Actl6a stability. This Fto/Actl6a/Sox2 axis is a promising therapeutic target for SCI repair.

## Highlights

• Actl6a enhances autophagy and inhibits neuronal apoptosis, promoting spinal cord injury (SCI) repair.
• Actl6a directly interacts with Sox2 to upregulate Atg5 and Atg7, exerting protective effects on neurons during SCI.
• Fto-mediated m6A methylation regulates Actl6a mRNA stability, emphasizing its role in injury response.

## Graphical abstract

Fto-Actl6a-Sox2 Pathway in SCI Recovery Fto regulates m6A methylation of Actl6a mRNA, influencing its stability and expression. Actl6a and Sox2 co-regulate the autophagy genes Atg5 and Atg7, promoting autophagy, reducing apoptosis, and enhancing neural cell survival post-SCI.

**Figure:**
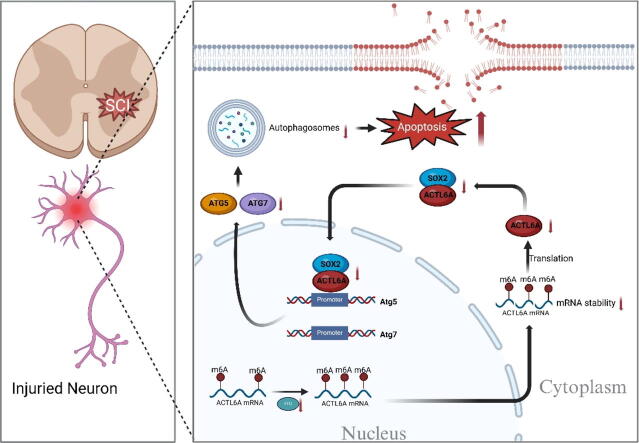

## Introduction

Spinal cord injury (SCI) is a severe central nervous system disorder, and despite decades of research, effective treatments for its repair remain elusive [1], [2]. Although significant advances have been made in SCI treatment, options that can meaningfully promote neuronal regeneration and functional recovery are still limited. Progress in research into the injury, pathogenesis, and treatment of SCI has led to a deeper understanding of the complex mechanisms involved in SCI. Studies have identified key secondary injury pathways, such as neuroinflammation, oxidative stress, and apoptosis, that exacerbate tissue damage following the initial mechanical trauma. This has shifted research towards targeting these secondary injury mechanisms to reduce damage and improve recovery. Additionally, advancements in neurobiology and regenerative medicine have provided new insights into potential therapeutic approaches, including the use of stem cell therapy, gene therapy, and neuroprotective agents. However, translating these promising treatments into effective clinical interventions remains a major challenge, and ongoing research continues to explore innovative ways to promote neuronal survival, axonal regeneration, and functional recovery after SCI.[3], [4], [5].

Actin-like protein 6A (Actl6a), also known as the 53 kDa BRG-1/human BRM-associated factor (BAF53a), plays a vital role in several cellular processes, including vesicle transport, spindle orientation, nuclear migration, cell cycle regulation, and chromatin remodeling[6], [7], [8], [9]. Its role in regulating cell fate has been validated across multiple cancers, highlighting its involvement in cell transformation, proliferation, and migration[10], [11], [12], [13], [14]. Studies have demonstrated that Actl6a regulates the recognition of axonal diameters by modulating myelinating cells in the peripheral nervous system, thereby influencing myelin thickness and axonal diameter. Additionally, Actl6a plays a key role in neuron-specific chromatin remodeling complexes, especially in regulating dendritic development. These complexes are essential for the morphological and functional growth of dendrites, reconfiguring gene expression in neurons and thereby influencing dendritic branching and the plasticity of neural networks[15], [16]. Despite the insights gained from oncology and neurobiology research, the specific role of Actl6a in neural protection and spinal cord injury repair remains insufficiently understood.

Sox2, a transcription factor crucial for maintaining the pluripotency of neural stem cells and regulating neural development, is integral to the regenerative capacity of neural cells and their response to injury[17], [18], [19], [20], [21]. Sox2 has been shown to play a critical role in myelination within the central nervous system, affecting both the proliferation of oligodendrocyte precursor cells and their differentiation into oligodendrocytes. While its role in neural development is well established, its specific functions in neural repair after SCI require further clarification[22], [23].

Autophagy plays a complex role in neural protection, facilitating the clearance of debris from dying neurons, such as damaged organelles and protein aggregates, thus reducing inflammation and protecting adjacent neurons[24], [25], [26]. Proper regulation of autophagy is essential for treating SCI, as it requires the precise control of autophagy-related gene expression and function[27], [28]. Atg5 and Atg7, two key regulatory proteins in the autophagy process, are critical for autophagosome formation and their subsequent fusion with lysosomes, essential steps in intracellular waste degradation[29]. The Atg5-Atg12 complex, along with Atg16L, facilitates autophagosome assembly, while Atg7 acts as an E1 enzyme in the ubiquitin-like modification process, which is vital for the maturation of these structures[30], [31], [32], [33].

In this study, we explore the mechanisms by which Actl6a and Sox2 regulate autophagy and their potential applications in neural protection following SCI. Our research identifies key roles for these factors in neural injury and repair, providing new therapeutic targets for SCI. Furthermore, we found that m6A (N6-methyladenosine) modification of Actl6a affects its expression. Our findings suggest that the Fto/Actl6a/Sox2 axis represents a promising target for SCI repair.

## Methods

### Primary neuron Isolation

Under sterile conditions, euthanize pregnant C57 via cervical dislocation, immerse them in ice-cold ethanol for 30 s, and dissect to extract the brain, rinsing it in cold phosphate-buffered saline (PBS). Using a dissecting microscope, remove the cerebral cortex and transfer it to a container with papain, incubating at 37 °C for 15–30 min to facilitate cell dissociation. After incubation, gently triturate the tissue using a cell strainer or pipette to create a single-cell suspension. Centrifuge to remove the enzymatic solution, resuspend the cells in a neuron-specific growth medium and seed them into dishes pre-coated with poly-D-lysine. Maintain the cultures in a 37 °C, 5 % CO_2_ incubator, replacing the medium regularly to ensure cell viability. The purity of the isolated primary neurons exceeded 80 %, as confirmed by immunofluorescence staining for the neuron-specific marker β-III-tubulin.

## Cell culture

For cell culture and gene function studies, use the HT-22 mouse hippocampal neuronal cell line (SCC129) and primary neurons isolated from the hippocampal region of neonatal mice. Cultivate HT-22 cells in Dulbecco’s Modified Eagle Medium (DMEM) supplemented with 10 % (v/v) fetal bovine serum (FBS) to provide essential growth factors. For primary neurons, use Neurobasal medium, supplemented with B27 and 2 mM L-glutamine. Induce oxidative stress in HT-22 cells with 200 µM H_2_O_2_ and in primary neurons with 400 µM H_2_O_2_[34], [35], adjusting the H_2_O_2_ concentration based on the cell type to ensure experimental consistency.

## Animal model construction

Experimental scheme of Rats was shown in S7.

Seven-week-old female SD rats were selected from Zhuhai Beston Biotechnology Co., (China, Zhuhai) due to their shorter urethras, which facilitate manual urination[36]. Rats were acclimated for seven days prior to the experiments to ensure they were in healthy condition.

The animals were divided into four groups with different numbers of rats:

Sham group underwent only laminectomy without spinal cord injury (n = 9);

SCI group underwent spinal cord injury without any gene intervention (n = 9);

SCI + Vector group received a vector injection following spinal cord injury (n = 9);

SCI + AAV (OE) group was injected with AAV vectors containing the Actl6a gene after spinal cord injury (n = 9).

Before initiating the spinal cord injury (SCI) model, we overexpressed Actl6a directly at the T9-T10 injury site (approximately 300 μm in depth) by injecting adeno-associated virus (AAV). Specifically, prior to establishing the SCI model, we injected AAV-Actl6a into the T9-T10 injury site, with a volume of 10 µL and a concentration of 1 × 10^12 viral genomes (vg). The injection was performed using a Hamilton syringe (Hamilton Co., USA) for precise manual injection at a flow rate of 200 nL/min, ensuring accurate delivery of the virus to the injury site. The injection took place before the SCI model was established, and 14 days were allowed for gene expression to stabilize. To verify the overexpression of Actl6a, we assessed Actl6a gene expression and protein levels via Western Blot (WB), real-time quantitative PCR (qPCR), and immunofluorescence (IF) techniques 14 days post-injection to ensure full expression of Actl6a.

After ensuring stable expression of Actl6a, we proceeded with the spinal cord injury (SCI) model. Anesthetize the rats with 1 % pentobarbital sodium (50 mg/kg body weight) and perform a standard laminectomy at T9-T10 to expose the dorsum spinal cord without damaging the dura mater. Induce injury using a spinal cord impactor (W.M. KECK+, USA), dropping a 5-gram weight from a height of 3 cm. Suture the incision with 4–0 silk thread, and house the rats in a temperature-controlled recovery room. Include a Sham group undergoing only laminectomy. Post-operatively, manually assist urinary evacuation three times daily and administer gentamicin sulfate (30 mg/kg intraperitoneally) for the first three days.

At the end of the study, euthanize the rats with an overdose of pentobarbital sodium. Perform behavioral and histological evaluations at predetermined intervals (days 3, 14, 21, and 28) to assess SCI effects and treatment efficacy. Additionally, spinal cord tissue will be harvested for Western Blot (WB) analysis to validate the expression of autophagy and apoptosis markers, further confirming the molecular effects of the treatment.

This study was conducted in accordance with the ethical standards of the National Research Council's Guide for the Care and Use of Laboratory Animals. All animal care and experimental protocols were reviewed and approved by the Experimental Animal Welfare Ethics Review Committee of the Second Affiliated Hospital of Guangzhou Medical University (Approval number: A2023—049).

## Autophagic flux assay

To determine the level of autophagy flux, HT22 cells were transfected with Ad-mCherry-GFP-LC3B (Beyotime, Shanghai, China) for 24 h. In this assay, intense red fluorescence from LC3 proteins indicated that autophagosomes had fused with lysosomes in the acidic microenvironment, forming autolysosomes, which reflects a smooth and active autophagic flux. In contrast, intense yellow fluorescence suggested that autophagosomes were unable to fuse with lysosomes, implying an inhibition of autophagy.

## Tissue section preparation and HE, Masson staining

On days 3 and 28 of the experiment, the mice were deeply anesthetized and perfused through the heart with phosphate-buffered saline (PBS, pH 7.4). Subsequently, 4 % (w/v) paraformaldehyde (PFA) was added for fixation. Next, spinal cord segments extending 4 mm rostrally from the epicenter were excised at a length of 1 mm, along with the entire segment (10 mm long, with the epicenter in the center), and these tissues were fixed in 4 % PFA for 24 h.

The fixed tissues were then dehydrated in a series of ethanol solutions of increasing concentrations. The dehydrated tissues were subsequently embedded in paraffin and oriented appropriately for sectioning. The embedded spinal cords were sliced into 5-µm sections using a microtome and mounted on gelatin-coated slides.

Histopathological examination was performed using Hematoxylin and Eosin (HE) staining to observe the longitudinal sections of the spinal cord. Masson's trichrome staining was also applied to longitudinal sections, which facilitated observation of collagen fiber changes critical for understanding the processes of inflammation and tissue repair. After staining, bright-field images were captured using a light microscope (Olympus, Japan) to conduct detailed analysis of the stained tissues.

## AAV construction

The AAV9 serotype was used due to its high transduction efficiency in the nervous system[37]. AAV-Actl6a, obtained from Obio Technology, was used to overexpress Actl6a in rats, with AAV vehicles serving as the control.

## Functional behavioral Evaluation

Hind limb motor function in rats was evaluated using the Basso, Beattie, and Bresnahan (BBB) scale and the inclined plane test at various time points. The BBB scale ranges from 0 to 21, where 0 indicates no observable hind limb movement and 21 represents normal motor function, assessing limb coordination and movement[38].

In the inclined plane test, rats were placed on a board that was gradually inclined until the rat could no longer maintain its position for 5 s. This test measures the maximum angle at which a rat can maintain its position, providing insight into muscular strength and balance.

Additionally, footprint analysis was conducted to compare the movement patterns of rats in different groups[39]. The forelimbs and hind limbs were dyed blue and red, respectively. Each rat was evaluated by two researchers who were blinded to the treatment conditions.

## Cell transfection

For overexpression of Actl6a, Sox2, and Fto, the pSLenti-EF1-P2A-Puro-CMV-MCS-3xFLAG-WPRE lentiviral vectors containing the full-length cDNA sequences (Actl6a: NM_019673.3, Sox2: NM_011443.4, Fto: NM_011936.2) were constructed by Obio Technology (Shanghai, China). An empty pSLenti-EF1-P2A-Puro-CMV-MCS-3xFLAG-WPRE vector was used as a control. The virus titer for overexpression vectors was 1 × 10^9 transducing units (TU)/mL. For knockdown experiments, the pSLenti-U6-shRNA-CMV-F2A-Puro-WPRE lentiviral vectors containing shRNA sequences targeting Actl6a, Sox2, and Fto were used. The specific shRNA sequences were as follows: Actl6a: GGACTGCCCTAAGGTTGATTT, Sox2: AGGAGCACCCGGATTATAAAT, and Fto: TTGAAAGAGGAGCCCTATTTC. The shRNA sequences for the knockdown experiments were synthesized by Obio Technology. The catalog number for the shRNA vectors is pSLenti-U6-shRNA-CMV-F2A-Puro-WPRE. The viral titer for knockdown vectors was 1 × 10^9 TU/mL, and transfection was carried out according to the manufacturer’s protocol. Cells were transduced with the lentivirus at a multiplicity of infection (MOI) of 10 to ensure efficient gene expression or knockdown. After 48 h, cells were selected with puromycin (P8833, Sigma-Aldrich) at 2 μg/mL for stable expression of overexpressed or knocked-down genes.

## Western blot and Co-Immunoprecipitation (CO-IP)

For Western blotting, cells or spinal cord samples were lysed using a buffer containing 0.1 % Triton X-100 (9002–93-1, Sigma-Aldrich), 50 mM Tris-Cl (pH 7.5), 0.1 % NP-40 (NP-40, Sigma-Aldrich), 150 mM NaCl, and 0.1 M EDTA, supplemented with phosphatase inhibitors (P8849, Sigma-Aldrich) and protease inhibitors (P5726, Sigma-Aldrich). The lysates were incubated at 4 °C for 30 min. Proteins were separated by SDS-PAGE, transferred to PVDF membranes (Millipore), and incubated with specific primary antibodies, followed by HRP-conjugated secondary antibodies (A0545, Sigma-Aldrich). Western Blot detection was carried out using enhanced chemiluminescence (ECL) reagents (32106, Thermo Fisher). The primary antibodies used in this study were Actl6a (1:1000 dilution, ab3882, Abcam), Sox2 (1:1000 dilution, ab171380, Abcam), Fto (1:1000 dilution, ab280081, Abcam), Caspase-3 (1:1000 dilution, ab32351, Abcam), Cleaved-Caspase-3 (1:1000 dilution, ab2302, Abcam), Bax (1:1000 dilution, ab32503, Abcam), Bcl-2 (1:1000 dilution, ab182858, Abcam), LC3 (1:1000 dilution, 13082, CST), p62 (1:1000 dilution, AF5384, Affinity), Atg5 (1:1000 dilution, ab108327, Abcam), Atg7 (1:1000 dilution, ab133528, Abcam), IgG (Rabbit, control) (1:5000 dilution, A-2094, Sigma-Aldrich), M6A (1:1000 dilution, 202003, Synaptic Systems), and GAPDH (1:2000 dilution, ab172730, Abcam). For Co-IP experiments, cell lysates were incubated overnight at 4 °C with specific antibodies targeting Actl6a (ab3882, Abcam) and Sox2 (ab171380, Abcam). After incubation, immune complexes were captured using Protein A/G beads (20421, Thermo Fisher) and analyzed by Western blot. The antibodies used for WB and Co-IP are listed in Table S2.

## m6A Dot blot assays

Total RNA was harvested as previously described, with RNA quantity monitored throughout the process. RNA was denatured by heating at 95 °C for 10 min, then immediately placed on ice. The mRNA samples were blotted onto an N + nylon membrane (FFN10, Beyotime, China), followed by ultraviolet cross-linking and blocking with 5 % nonfat milk in PBST for one hour at room temperature. The membranes were incubated overnight at 4 °C with an m6A-specific antibody (1:5000, 202003, Synaptic Systems). After washing, the membranes were incubated with a goat anti-rabbit IgG HRP antibody (bs-40295G-HRP, Bioss) with gentle shaking for 1 h at room temperature. Membranes were washed again, treated with an ECL detection reagent (MA0186, Meilunbio, China), and visualized using a detection system.

## Quantitative Real-Time PCR (qPCR)

Total RNA was extracted using TRIzol reagent (Invitrogen) and reverse transcribed using ReverTra Ace® qPCR RT Master Mix with gDNA Remover (TOYOBO), according to the manufacturer’s instructions. Gene expression was quantified using the LightCycler 480 PCR system (Roche) and 2 × SYBR Green qPCR Master Mix (biotool, #B21203), with GAPDH serving as the reference gene. Primer sequences are listed in Table S1.

## Cell viability assay

Cell viability was assessed using the Cell Counting Kit-8 (CCK-8, Yeasen Biotech). HT22 cells (5 × 10^3^ cells per well) were seeded in a 96-well plate and allowed to adhere overnight in complete culture medium. At designated time points (0, 3, 6, 12, 18, and 24 h), 10 μL of CCK-8 solution was added to each well, and the cells were incubated at 37 °C in a humidified incubator for 2 h. The absorbance was measured at 450 nm using a microplate reader (SynergyH1, BIOTEK). To ensure reliable results, each group was tested in triplicate, and the experiment was repeated at least three times.

## RNA immunoprecipitation (RIP) assay

The EZ-Magna RIP Kit (Millipore, 17–700, Billerica, MA) was used to perform RIP assays. Cell lysates were mixed with RIP buffer containing specific antibodies against Fto (1:2500 dilution, ab280081, Abcam), with IgG (A-2094, Sigma-Aldrich, 1:1000 dilution) serving as a negative control. After incubation with magnetic beads (20421, Thermo Fisher) for 4 h at 4 °C, the beads were washed, and the cell lysate-protein-antibody complexes were digested with 0.1 % SDS and 0.5 mg/mL proteinase K (25530031, Thermo Fisher) at 55 °C for 30 min. Co-precipitated RNA was then analyzed via qRT-PCR using specific primers listed in Table S1.

## MeRIP assay

Total RNA was extracted using RNAiso Plus (Takara, Japan), with DNase treatment to remove DNA contamination. m6A-containing RNA fragments were immunoprecipitated using m6A antibodies linked to magnetic beads. A cleavage enzyme mix was applied to trim the RNA sequences flanking the m6A sites. After elution, purification, and release of enriched RNA, qRT-PCR was used to quantify changes in m6A methylation of target genes.

## Chromatin immunoprecipitation (ChIP) assay

ChIP assays were performed using a modified fast ChIP protocol. Cells were fixed with 1 % formaldehyde (F8775, Sigma-Aldrich) at room temperature for 15 min. Sheared chromatin was incubated overnight at 4 °C with specific antibodies under gentle rotation. The following primary antibodies were used: Actl6a (1:5000 dilution, ab3882, Abcam), Sox2 (1:8000 dilution, ab171380, Abcam). Immunoprecipitates were then mixed with Protein A agarose beads (sc2003, Santa Cruz Biotechnology) and rotated for 2 h at 4 °C. To reverse cross-links, Chelex 100 resin (1421253, Bio-Rad) was added to both immunoprecipitates and input DNA samples and incubated at 100 °C for 10 min. After proteinase K treatment (25530031, Thermo Fisher) for 30 min, the recovered DNA was analyzed via qPCR using primers listed in Table S1.

## Immunofluorescence (IF) staining

Cells were placed in six-well plates and treated accordingly. After treatment, cells were fixed with 3.7 % formaldehyde for 10 min at room temperature. Permeabilization was performed using 1 % SDS in PBS for 2 min, followed by blocking with 1 % BSA in PBS for 30 min. Primary antibodies were incubated overnight at 4 °C, followed by a 1-hour incubation with secondary antibodies. Nuclear staining was achieved using DAPI. Images were captured using an Olympus IX83 microscope at 40 × magnification and analyzed using FIJI software.

The transverse and longitudinal sections of spinal cord tissue were prepared at 5-µm thickness and subjected to immunofluorescence staining, as described above. The tissue samples from all groups were deparaffinized, rehydrated, and washed. They were then placed in a 10.2 mM sodium citrate buffer solution and incubated at 95 °C for 20 min for antigen retrieval. Permeabilization was achieved using 0.1 % PBS-Triton X-100 for 10 min, followed by blocking with 10 % goat serum in PBS for 1 h. Primary antibodies were applied overnight at 4 °C. The next day, sections were incubated with secondary antibodies at 37 °C for 1 h and then counterstained with DAPI. Images were captured using an Olympus IX83 microscope for detailed analysis.

## TUNEL staining

For tissue sections and cultured cells, samples were fixed with 4 % formaldehyde. Tissue sections were embedded in paraffin and sectioned, while cells were fixed directly on slides. TUNEL staining was performed according to the manufacturer’s instructions (C1088, Beyotime) to detect DNA breaks, identifying apoptotic cells. Nuclei were counterstained with DAPI, and images were captured using a fluorescence microscope. Image analysis software quantified TUNEL-positive cells to assess apoptosis levels.

## Transmission electron microscopy (TEM)

For TEM analysis, treated or untreated cells were washed, digested with trypsin, and resuspended. Cells were then fixed with 2.5 % glutaraldehyde for autophagosome analysis by transmission electron microscopy. Images were captured using a JEM-1400 PLUS microscope. To ensure consistency, three cells per group were selected for autophagosome analysis.

For spinal cord tissue samples, the tissues were fixed in a glutaraldehyde-based fixative to preserve cellular integrity, dehydrated, and embedded in resin. Ultra-thin sections were cut, mounted on copper grids, and stained with uranyl acetate and lead citrate to enhance structural contrast under the electron microscope. Autophagosome morphology and quantity were analyzed by examining the number of autophagosomes in three randomly selected cells per group.

## Dual-Luciferase reporter assay

Promoter regions and mutation sites of Atg5 and Atg7 were cloned downstream of the pmirGLO dual-luciferase reporter vector (Obio Technology, Shanghai, China). Wild-type and mutant Atg5/Atg7 luciferase plasmids (Fig. 6F) were co-transfected with other plasmids. Luciferase activity was measured using a dual-luciferase reporter assay system (Promega, Madison, USA), with firefly luciferase activity normalized to Renilla luciferase activity.

## Transcriptome sequencing (RNA-seq)

RNA-seq was conducted by Cloud-Seq Biotech (Shanghai, China). rRNAs were removed from total RNA before library construction. Quality control and quantification of the library were performed, and sequencing was carried out on an Illumina HiSeq instrument using a 150 bp paired-end mode. After sequencing, raw data were processed and aligned to the reference genome using STAR aligner.

## Gene ontology (GO) analysis

To gain insight into the functional significance of differentially expressed genes (DEGs), Gene Ontology (GO) analysis was performed using the DAVID bioinformatics resources (https://david.ncifcrf.gov/). GO terms related to biological processes, molecular functions, and cellular components were identified. A p-value threshold of < 0.05 was used to define significantly enriched GO terms. Enriched pathways and molecular functions were visualized in bar plots and bubble charts.

## RNA degradation

Cells were treated with 5 µg/ml actinomycin D (Sigma-Aldrich, A9415) to inhibit global mRNA transcription. At designated time points, cells were collected, and RNA was extracted for reverse transcription. qPCR was used to measure mRNA transcription levels.

## Statistical analyses

Statistical analyses were performed using GraphPad Prism 8.0 (GraphPad Software, La Jolla, CA). Data are presented as mean ± standard deviation (mean ± SD) from at least three independent experiments. For comparisons between two groups, an unpaired two-tailed Student’s *t*-test was used. For comparisons involving more than two groups, one-way or two-way analysis of variance (ANOVA) was performed, followed by Tukey’s post hoc test for pairwise group comparisons. For the CCK-8 assay, where repeated measurements were taken from the same cells at different time points, repeated measures ANOVA was applied to account for within-group correlation over time. If normality was not met, nonparametric tests such as the Friedman test were used. Statistical significance was set at P < 0.05.

## Results

**Downregulation of Actl6a in spinal cord injury**

**Fig. 1:**
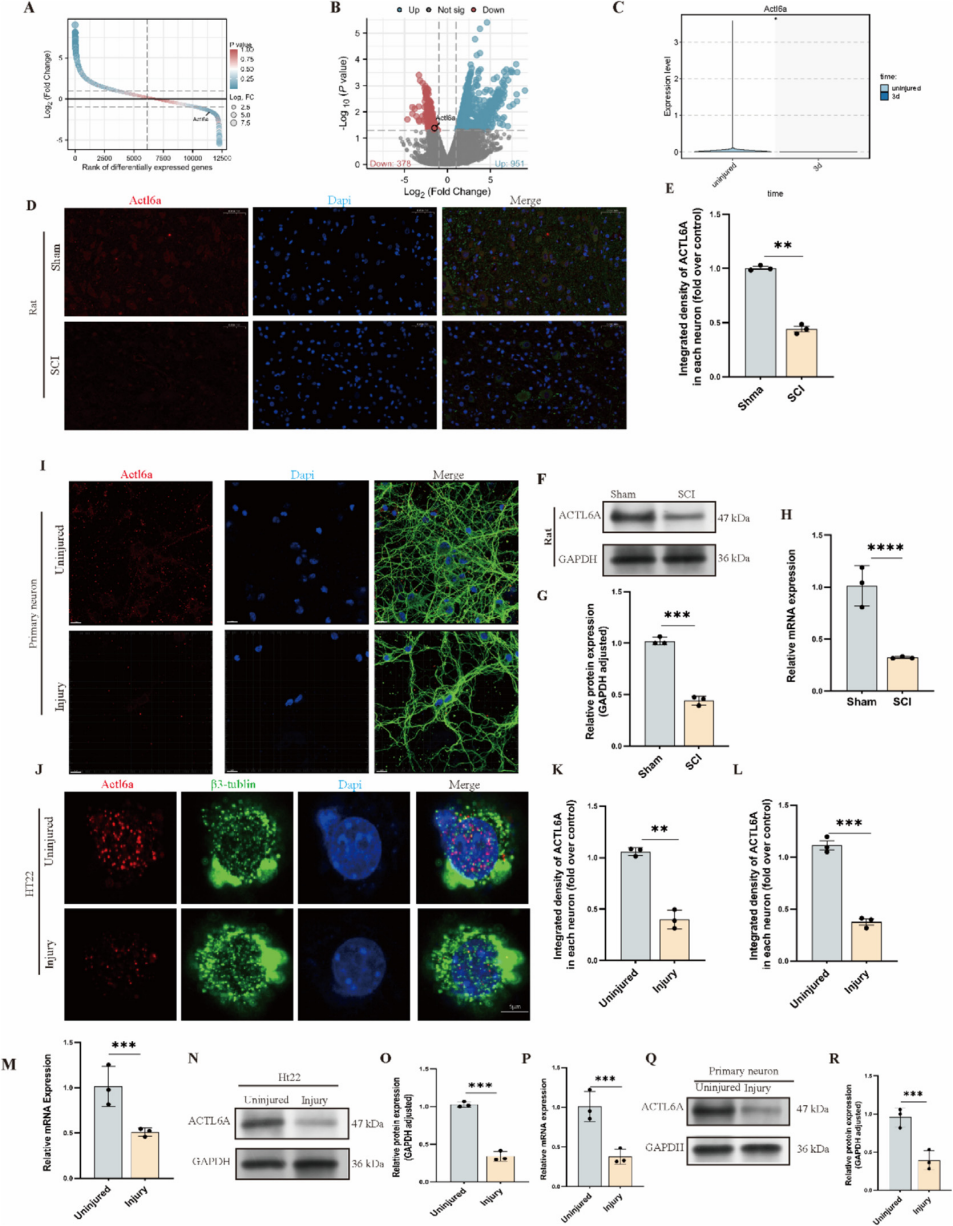
Analysis of Actl6a Expression in Various Neuronal Injury Models (A-B) RNA sequencing shows Actl6a expression differences between control and injury groups. (C) Single-cell RNA-seq reveals Actl6a expression changes in neurons at three days post-SCI compared to sham treatment. (D-E) IF showing Actl6a protein expression of the spinal cord ventral horn grey matter in the indicated groups on day 3 after SCI; scale bar: 50 μm. The data are presented as the means ± SEMs (n = 3 per group); (F-G) Western blot of Actl6a protein expression in spinal cord lesions from the indicated groups on day 3 after SCI. (H) qPCR showing Actl6a mRNA expression in spinal cord lesions from the indicated groups on day 3 after SCI. (I-J) IF of Actl6a in HT22 cells post-injury; scale bar: 5 μm. (K) qPCR analysis of Actl6a mRNA in primary neurons derived from injured tissue. (L-M) Western blot analysis of Actl6a protein levels in primary neurons with and without injury; GAPDH is used as a loading control. (N) qPCR measures Actl6a mRNA levels in HT22 cells post-injury. (O-P) Western blot of Actl6a protein expression in primary neurons post-injury. (Q-R) Immunofluorescence (IF) staining of Actl6a protein localization and expression in primary neurons post-injury; scale bar: 15 μm. *P < 0.05, **P < 0.01, and ***P < 0.001 indicate significant differences; ns, not significant. Significance was calculated using an independent samples *t*-test to compare the two groups.

Our transcriptomic analysis comparing RNA-seq data between the SCI group and control group revealed a significant downregulation of Actl6a in the SCI group (Fig. 1A-B). Complementary single-cell sequencing data from public repositories[40] further confirmed decreased Actl6a expression within neurons, implicating its critical involvement in the biological response to SCI (Fig. 1C). To investigate this further, we established an SCI animal model and performed sampling at three days post-injury. Immunofluorescence (IF) assays showed a marked reduction in Actl6a expression in the SCI group relative to controls, consistent with our bioinformatic analysis (Fig. 1D-E). These findings were corroborated by Western blot (WB) and quantitative PCR (qPCR), confirming the reduction in Actl6a expression, thus suggesting its regulatory role in SCI (Fig. 1F-H).

Further validation was carried out using two cellular models: primary neurons and the HT22 cell line. Both models were subjected to hydrogen peroxide (H_2_O_2_)-induced oxidative stress. WB, qPCR, and IF staining revealed a significant decrease in Actl6a expression under oxidative stress, consistent with in vivo findings, and indicated changes in its cellular localization in both models (Fig. 1I-R).

To investigate whether the Actl6a response in spinal cord injury (SCI) is shared by all neurons or whether some specific populations (including non-neuronal components) are particularly vulnerable, we performed single-cell RNA sequencing analysis. We classified the cell populations into neuronal cells, including neurons (neuron), oligodendrocytes (ODC), and oligodendrocyte precursor cells (OPC), and supporting cells, including astrocytes (astrocyte), microglia (microglia), endothelial cells (endothelial), pericytes (pericyte), stromal cells (stromal), and ependymal cells (ependyma). The analysis revealed that Actl6a expression was lowest in neurons, indicating that neurons are likely the most affected following spinal cord injury. (SF1 A).

Subsequently, we further classified the neuronal population into motor neurons (motor neurons), excitatory interneurons (excitatory interneurons), inhibitory interneurons (inhibitory interneurons), and other spinal cord neurons (other spinal cord neurons). Notably, there were no significant differences in Actl6a expression among these neuronal subtypes, suggesting that Actl6a’s role in spinal cord injury may be widely shared across different types of neurons. (SF1 B).

## Actl6a Aids functional recovery after spinal cord injury

**Fig. 2:**
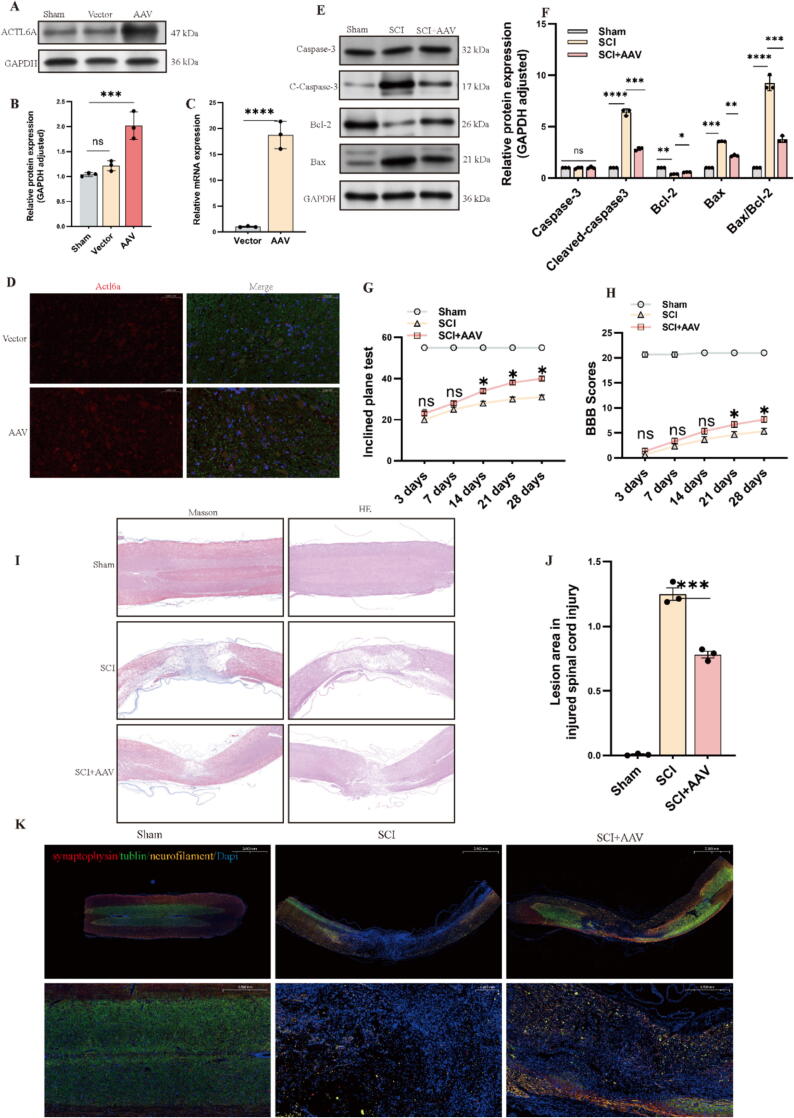
Analysis of Actl6a Expression and Functional Outcomes After AAV Injection and Spinal Cord Injury in Rats. (A-B) Western blot analysis of Actl6a protein in rat spinal cord 14 days post-AAV injection. (C) qPCR analysis of Actl6a mRNA expression 14 days post-AAV injection. (D) IF analysis of Actl6a expression in rat spinal cord 14 days post-AAV injection; scale bar: 50 μm. (E-F) Western blot analysis of apoptotic proteins in the injured spinal cord in spinal cord lesions from the indicated groups on day 3 after SCI. (G-H) Functional assessment using inclined plane test(G) and BBB scoring(H) at various time points post-injury. (I-J) Longitudinal spinal cord sections obtained from the groups on day 28 after SCI were examined by performing HE staining and Masson staining. scale bar: 1,000 μm (K) IF analysis for neuronal markers (Neurofilament, Tubulin, Synaptophysin) in longitudinal spinal cord sections obtained from the groups on day 28 after SCI; scale bar: 2 mm/0.2 mm. The data are presented as the means ± SDs (n = 3 per group);*P < 0.05, **P < 0.01, and ***P < 0.001 indicate significant differences; ns, not significant. Significance was calculated using two-way ANOVA followed by Tukey's multiple comparison test or independent samples *t*-test.

To explore the role of Actl6a, we induced its overexpression by injecting AAV-Actl6a 14 days before initiating the SCI model, ensuring elevated gene expression at the time of injury. The success of overexpression was confirmed by WB, qPCR, and IF staining (Fig. 2A-D). TUNEL staining revealed a substantial reduction in apoptosis in the SCI + AAV-Actl6a group (S5A), supported by WB data showing decreased levels of cleaved-Caspase-3 and Bax, alongside increased Bcl-2 expression (Fig. 2E, F).

Following SCI, functional assessments—including BBB scoring, inclined plane tests, and CatWalk automated gait analysis—were performed (Fig. 2G, H and S6). Results showed significant improvements in neurological function in the SCI + AAV-Actl6a group compared to the SCI-only group.

Histological analyses using HE and Masson staining indicated a notably reduced area of damage in the AAV-Actl6a group, suggesting enhanced tissue protection (Fig. 2I, J). IF staining of Neurofilament, Tubulin, and Synaptophysin further demonstrated improved neuronal integrity and synaptic structure (Fig. 2K).

## Actl6a expression Inhibits cell apoptosis

**Fig. 3:**
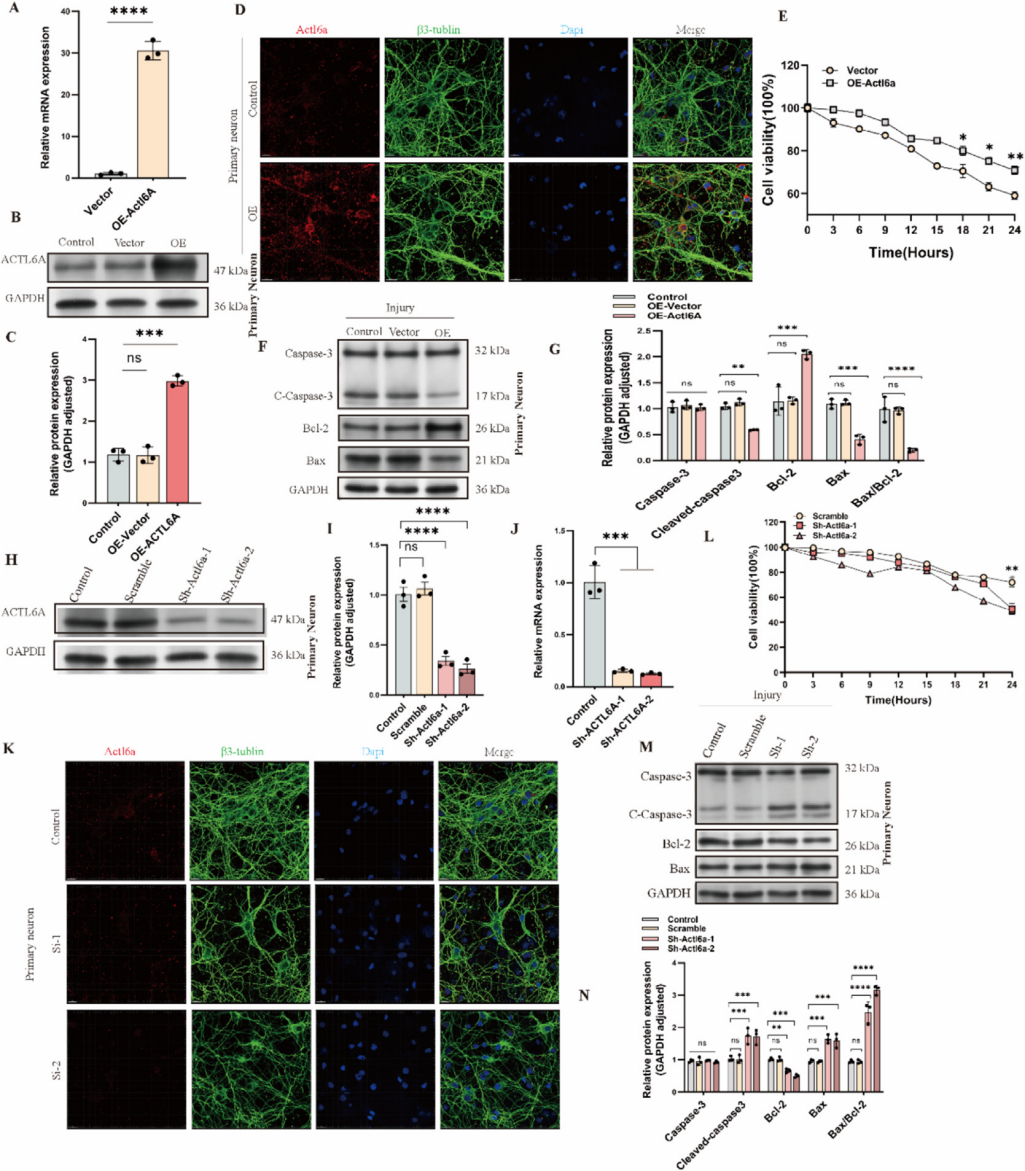
Actl6a Modulation Alters Apoptotic and Proliferative Responses (A) qPCR analysis of Actl6a mRNA levels after overexpression in primary neurons. (B-C) Western blot of Actl6a protein after overexpression in primary neurons; GAPDH as a loading control. (D) IF visualization of Actl6a following overexpression in primary neurons; scale bar: 15 μm. (E) CCK-8 assay to measure HT22 cell viability comparing control and Actl6a overexpression. (F-G) Western blot of apoptosis-related proteins after Actl6a overexpression; (G) shows quantification. (H-I) Western blot of Actl6a protein after knockdown in primary neurons; GAPDH as a control. (J) qPCR of Actl6a mRNA levels after knockdown in primary neurons. (K) IF showing Actl6a expression after knockdown in primary neurons; scale bar: 15 μm. (L) CCK-8 assay of cell viability in HT22 cells after Actl6a knockdown. (M−N) Western blot of apoptosis-related proteins after Actl6a knockdown in primary neurons. The data are presented as the means ± SDs (n = 3 per group);*P < 0.05, **P < 0.01, and ***P < 0.001 indicate significant differences; ns, not significant. Significance was calculated using two-way ANOVA followed by Tukey's multiple comparison test or independent samples *t*-test.

Following a detailed analysis of the role of Actl6a in SCI, we examined its effect on neuronal function in vitro. Both HT22 cells and primary neurons were used to manipulate Actl6a expression through knockdown (KD) and overexpression (OE). Validation of these genetic modifications was achieved using WB, qPCR, and IF assays (Fig. 3A-D, H-K and S1 C–H, M−P). Further experiments revealed that Actl6a KD significantly reduced cell proliferation, whereas OE enhanced it, highlighting Actl6a's critical role in regulating neuronal proliferation (Fig. 3E, L).

TUNEL assays showed increased apoptosis in the Actl6a KD group and decreased apoptosis in the OE group (S5B, C). WB analysis confirmed that KD led to higher levels of pro-apoptotic proteins (cleaved-Caspase-3 and Bax) and lower levels of anti-apoptotic Bcl-2. Conversely, OE resulted in reduced cleaved-Caspase-3 and Bax, and increased Bcl-2 expression (Fig. 3F, G, M, N and S1 I-L).

These in vitro findings suggest that Actl6a plays a significant role in regulating neuronal proliferation, apoptosis, migration, and structural integrity.

## Actl6a Alleviates apoptosis by enhancing autophagy

**Fig. 4:**
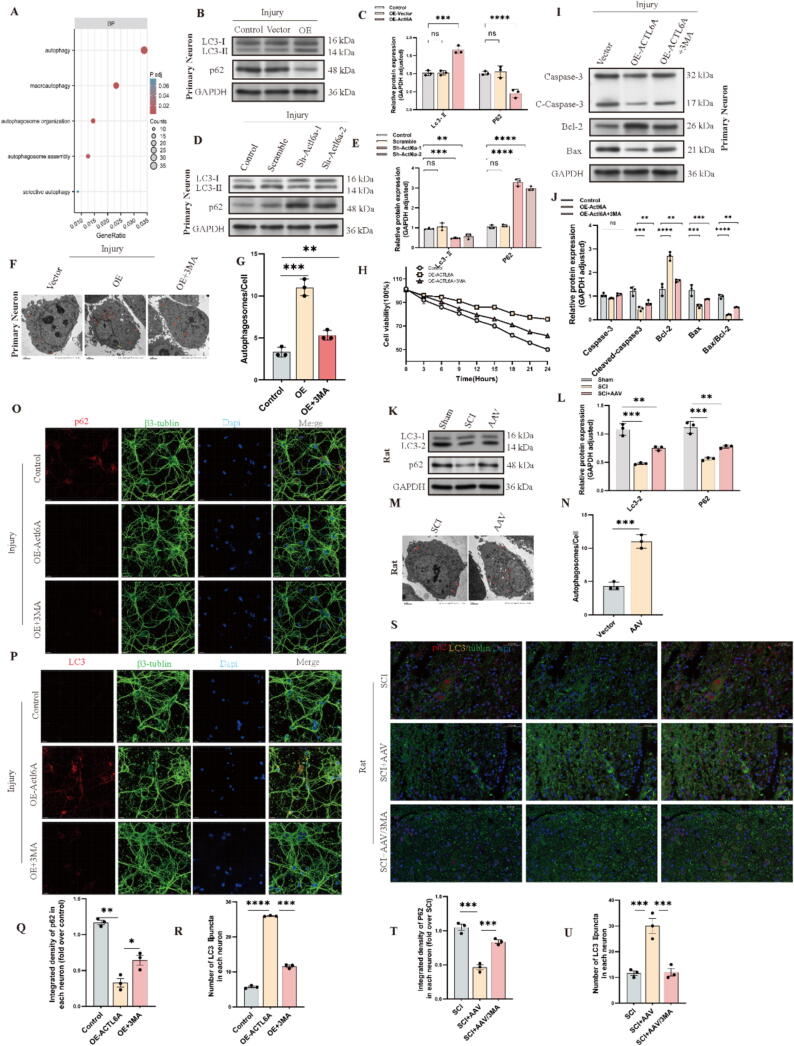
Actl6a Enhances Autophagy to Alleviate Apoptosis in Neurons and Rat Spinal Cord Models (A) GO analysis highlights pathways affected by neuronal injury. (B-C) Western blot of Lc3-I, Lc3-II, and p62 in primary neurons post-Actl6a overexpression, with quantification in (C). (D-E) Western blot of Lc3-I, Lc3-II, and p62 after Actl6a knockdown, with quantification in (E). (F-G) TEM images showing autophagosomes in primary neurons with control, OE, and OE + 3-MA groups, with quantification in (G). (H) CCK-8 assay of cell viability in HT22 cell lines with control, OE, and OE + 3-MA groups. (I-J) Western blot of apoptosis-related proteins in primary neurons from control, OE, and OE + 3-MA groups, with quantification in (J). (K-L) Western blot of autophagy markers in the injured spinal cord in spinal cord lesions from the indicated groups on day 3 after SCI, with quantification in (L). (M−N) TEM shows autophagosomes in the injured spinal cord in spinal cord lesions from the indicated groups on day 3 after SCI, with quantification in (N). (O-Q) IF to detect p62 changes in primary neurons post-Actl6a overexpression, with quantification in (Q);scale bar: 15 μm. (P-R) IF of LC3 in primary neurons post-Actl6a overexpression, with quantification in (R);scale bar: 15 μm. (S-U) IF of p62 and LC3 in rat spinal cord post-SCI, with quantification in (T) and (U) scale bar: 50 μm. The data are presented as the means ± SDs (n = 3 per group); *P < 0.05, **P < 0.01, and ***P < 0.001 indicate significant differences; ns, not significant. Significance was calculated using two-way ANOVA followed by Tukey's multiple comparison test or independent samples *t*-test.

In further investigation of Actl6a's role in apoptosis, we assessed its potential to mitigate apoptosis via autophagy. Gene ontology (GO) analysis revealed significant enrichment of autophagy pathways (Fig. 4A). Subsequent experiments modulated Actl6a expression through KD and OE. WB analyses of autophagy markers Lc3-I, Lc3-II, and p62 supported our hypothesis: Actl6a KD inhibited autophagy, as indicated by a reduced Lc3-II/Lc3-I ratio and elevated p62 levels, while OE enhanced autophagy, reflected by an increased Lc3-II/Lc3-I ratio and reduced p62 (Fig. 4B-E, O-R and S2A-D, I-L). Transmission electron microscopy (TEM) confirmed these findings, showing increased autophagosome formation in cells overexpressing Actl6a (Fig. 4F, G and S2G, H).

Further validation was obtained in an animal model, where the SCI + AAV-Actl6a group demonstrated heightened autophagy activity compared to the SCI group (Fig. 4K, L and S-U), as shown by autophagosome quantification via electron microscopy (Fig. 4M, N). To explore the impact of autophagy on cell survival, we conducted a rescue experiment using the autophagy inhibitor 3-MA[41], [42].. This experiment involved three groups: Control, OE-Actl6a, and OE-Actl6a treated with 3-MA (OE-Actl6a + 3MA). Inhibition of autophagy significantly reduced proliferation in the OE-Actl6a + 3MA group (Fig. 4H), and WB analysis of apoptosis markers revealed enhanced apoptosis, with increased levels of Caspase-3, cleaved-Caspase-3, and Bax, and decreased Bcl-2 (Fig. 4I, J and S2E, F).

To assess autophagic flux, we used the pCMV-mCherry-GFP-LC3B dual-label probe, and the results showed that Actl6a overexpression significantly enhanced yellow fluorescence, indicating increased autophagosome-lysosome fusion (S2F M). To further confirm whether the elevation of LC3-II was due to enhanced autophagic flux or impaired lysosomal degradation, we added Bafilomycin A, a lysosomal inhibitor, to our experimental groups[43]. The results showed that LC3-II levels were significantly higher in the Actl6a overexpression group when Bafilomycin A was introduced, confirming that the elevation of LC3-II was primarily due to enhanced autophagic flux rather than reduced lysosomal degradation (S2F N). These findings provide strong evidence for Actl6a's role in promoting autophagic flux.

These results confirm that Actl6a modulates apoptosis by promoting autophagy, emphasizing the crucial role of Actl6a in cellular protection. The diminished protective effect of Actl6a in the presence of 3-MA highlights the importance of autophagy in cell survival under stress.

## Actl6a regulates autophagy through Sox2

Actl6a is known to function as a transcriptional co-activator, influencing gene expression through the modulation of transcription factor (TF) activity[44]. As part of the SWI/SNF complex, ACTL6A can interact with a range of transcription factors to regulate various cellular processes, including autophagy. Building on our findings that Actl6a reduces cell apoptosis by enhancing autophagy, we further examined the pathways through which Actl6a influences autophagy. Using BioGRID[45] to identify potential interactions with transcription factors involved in autophagy pathways, we performed co-immunoprecipitation (Co-IP) experiments targeting various transcription factors. The results revealed a specific and significant interaction between Actl6a and Sox2 (Fig. 6D), while no conclusive interactions were observed with other transcription factors.

**Fig. 5:**
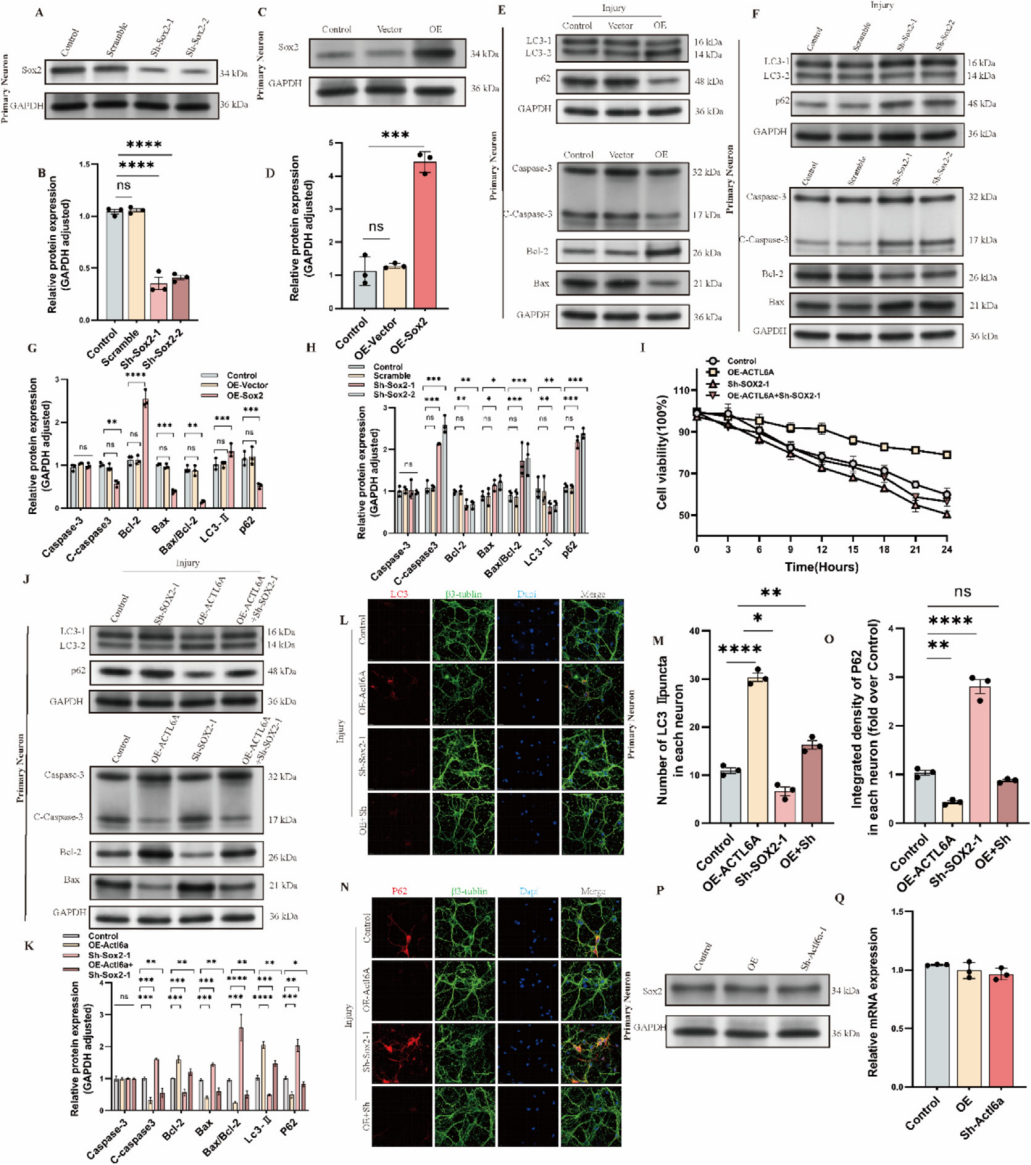
Sox2 and Actl6a Modulate Autophagy and Apoptosis (A-B) Western blot of Sox2 protein post-knockdown in primary neurons, with quantification in (B). (C-D) Western blot of Sox2 protein post-overexpression, with quantification in (D). (E, G) Western blot of autophagy and apoptosis proteins post-Sox2 overexpression, with quantification in (G). (F, H) Western blot of autophagy and apoptosis proteins post-Sox2 knockdown, with quantification in (H). (I) CCK-8 assay in HT22 cells under control, OE-Actl6a, Sh-Sox2, and OE-Actl6a + Sh-Sox2 groups. (J-K) Western blot of autophagy and apoptosis proteins in primary neurons under OE-Actl6a and Sh-Sox2 groups, with quantification in (K). (L-M) IF of LC3 in primary neurons under various conditions, with quantification in (M);scale bar: 15 μm. (N-O) IF of P62 in primary neurons, with quantification in (O);scale bar: 15 μm. (P) Western blot of Sox2 protein post-Actl6a modulation. (Q) qPCR of Sox2 mRNA post-Actl6a modulation. The data are presented as the means ± SDs (n = 3 per group); *P < 0.05, **P < 0.01, and ***P < 0.001 indicate significant differences; ns, not significant. Significance was calculated using two-way ANOVA followed by Tukey's multiple comparison test or independent samples *t*-test.

In vitro experiments using Sox2 knockdown (KD) and overexpression (OE) strategies showed that Sox2 KD increased apoptosis, while Sox2 OE reduced apoptosis (S5F, G). Western blot (WB) analyses of autophagy and apoptosis markers indicated that Sox2 OE increased the Lc3-II/Lc3-I ratio and decreased p62 levels, promoting autophagy and reducing apoptotic markers such as cleaved-Caspase-3 and Bax, while enhancing Bcl-2 expression. Conversely, Sox2 KD reduced the Lc3-II/Lc3-I ratio and increased p62, indicating suppressed autophagy and enhanced apoptosis (Fig. 5E-H).

**Fig. 6:**
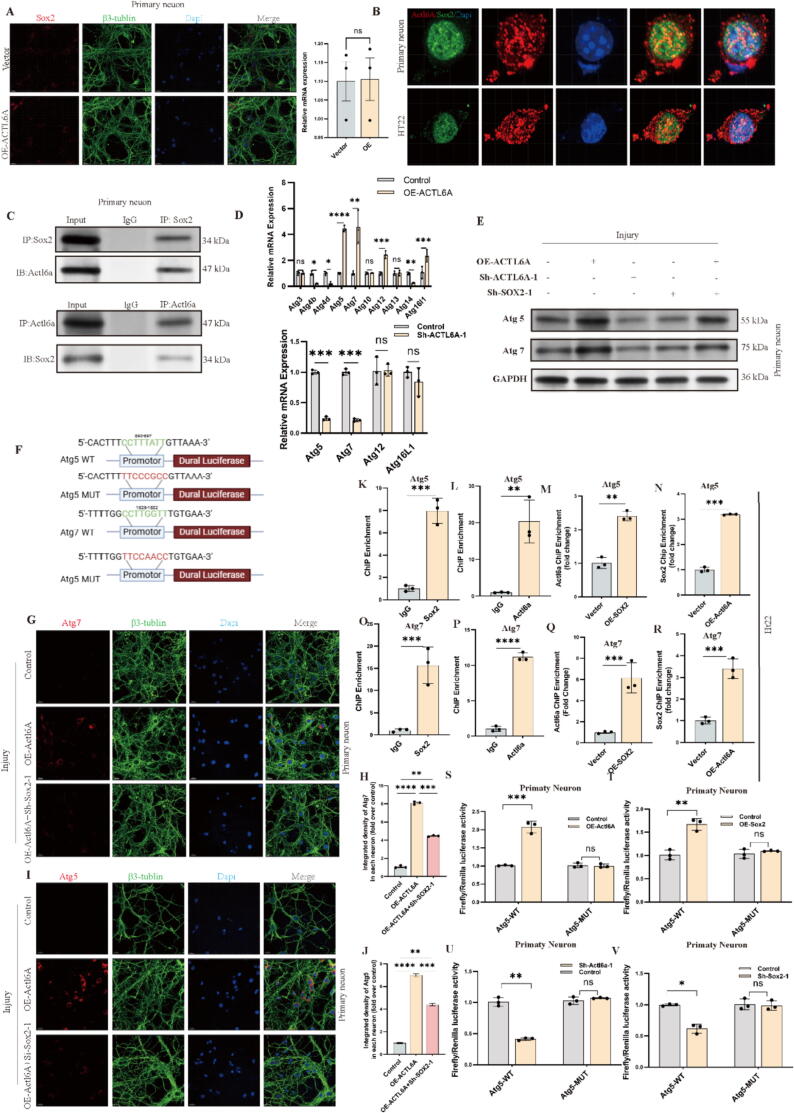
Actl6a and Sox2 Interactions Regulate Autophagy Genes. (A) IF showing Sox2 expression post-Actl6a overexpression in primary neurons; scale bar: 15 μm. (B) IF to visualize Actl6a and Sox2 colocalization in primary neurons and HT22, scale bar: 5 μm. (C) Co-IP assay to detect Actl6a-Sox2 interaction in primary neurons. (D) qPCR analysis of autophagy-related genes post-Actl6a overexpression and knockdown in primary neurons. (E) Western blot of Atg5 and Atg7 post-Actl6a and Sox2 modulation in primary neurons. (F) Schematic diagram of dualluciferase reporter constructs. (G-H) IF of Atg7 in primary neurons post-Actl6a modulation, with quantification in (H); scale bar: 15 μm. (I-J) IF of Atg5 post-Actl6a modulation, with quantification in (J). (K-R) ChIP assays of Actl6a and Sox2 binding to Atg5 and Atg7 promoters. (S-V) Luciferase assays to measure promoter activity of Atg5 and Atg7 with Actl6a or Sox2 modulation. The data are presented as the means ± SDs (n = 3 per group); *P < 0.05, **P < 0.01, and ***P < 0.001 indicate significant differences; ns, not significant. Significance was calculated using two-way ANOVA followed by Tukey's multiple comparison test or independent samples *t*-test.

A rescue experiment involving Actl6a overexpression and Sox2 KD further assessed these interactions. The results showed that while Actl6a overexpression alone enhanced autophagy and reduced apoptosis (Fig. 5J-O and S3I-L), the beneficial effects were diminished when Sox2 was knocked down, leading to decreased cell viability (Fig. 5I). These findings highlight Sox2′s essential role in mediating Actl6a's effects on autophagy, apoptosis, and cell viability, emphasizing the synergistic action between Actl6a and Sox2 in regulating autophagy.

## Actl6a influences the expression of Atg genes through Sox2

Our investigations revealed that Actl6a does not regulate autophagy by altering Sox2 expression levels (Fig. 5P,Q, 6A, and S4A). Instead, Actl6a affects Sox2′s ability to control the expression of specific autophagy-related genes. Quantitative PCR (qPCR) analysis showed that Actl6a overexpression significantly upregulated Atg5, Atg7, Atg12, and Atg16L1, while other autophagy-related genes, such as Atg3, Atg4b, and Atg14, were unaffected or downregulated (Fig. 6D and S4B). Knockdown of Actl6a led to reduced expression of Atg5 and Atg7 (Fig. 6D and S4C).

Western blot (WB) and immunofluorescence (IF) assays confirmed these findings, showing consistent trends for Atg5 and Atg7 expression (Fig. 6E, G-J, and S4D, E, F). Co-IP and IF assays further revealed that Actl6a physically interacts and colocalizes with Sox2 at the protein level, despite not altering Sox2′s mRNA or protein levels (Fig. 6B,C).

Chromatin immunoprecipitation (ChIP) assays targeting the promoter regions of Atg5 and Atg7 demonstrated that both Actl6a and Sox2 bind to these promoters, enhancing their interaction under overexpression conditions (Fig. 6K-R). Luciferase reporter assays with promoter mutations confirmed that the regulatory effects of Actl6a and Sox2 depend on their direct binding to specific promoter regions (Fig. 6S-V and S4G-R).

These findings illustrate that Actl6a interacts with Sox2 to regulate the expression of critical autophagy genes, Atg5 and Atg7, through promoter-level regulation, offering new insights into autophagy regulation and potential therapeutic targets.

## Fto influences Actl6a expression through m6A

**Fig. 7:**
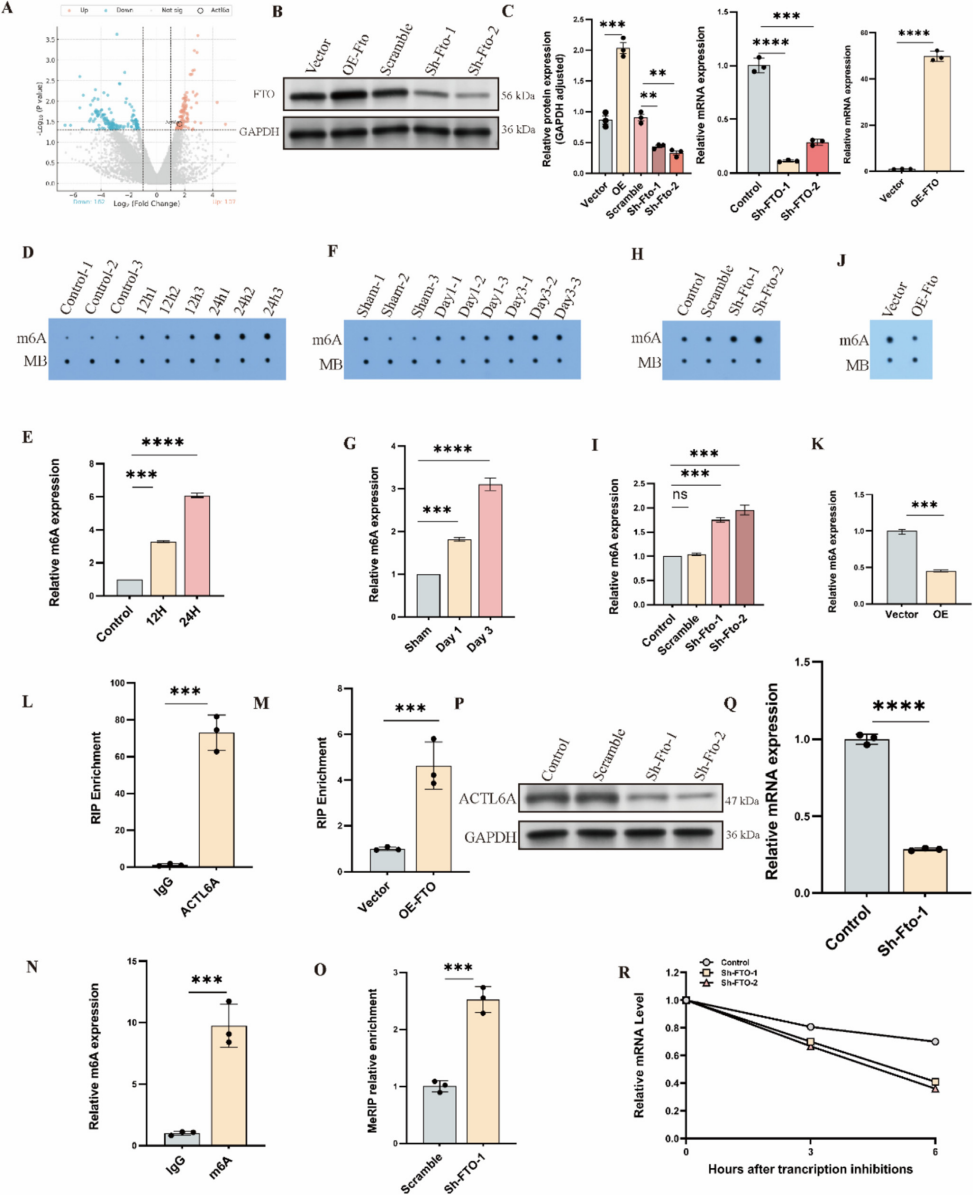
Fto Modulates Actl6a Expression via m6A Methylation. (A) RNA-seq analysis shows changes in Actl6a expression after STM2457 treatment. (B-C) Western blot of Actl6a post-FTO knockdown and overexpression, with quantification in (C). (D-G) Dot blot assays measure m6A levels post-injury and post-FTO modulation, with quantification. (H-I) Dot blot of m6A levels post-FTO knockdown, with quantification in (I). (J-K) Dot blot of m6A levels post-FTO overexpression, with quantification in (K). (L-M) RIP assays confirm FTO-Actl6a mRNA interaction. (N-O) MeRIP-qPCR measures m6A enrichment on Actl6a mRNA post-FTO modulation. (P-Q) Western blot of Actl6a protein post-FTO knockdown, with quantification in (Q). (R) Actl6a mRNA stability analysis post-FTO knockdown. The data are presented as the means ± SDs (n = 3 per group); *P < 0.05, **P < 0.01, and ***P < 0.001 indicate significant differences; ns, not significant. Significance was calculated using two-way ANOVA followed by Tukey's multiple comparison test or independent samples *t*-test.

We next investigated the role of epigenetic modifications, particularly m6A methylation, in regulating autophagy and cell fate post-spinal cord injury. Recent studies have shown that FTO demethylates m6A sites on Atg5 and Atg7 mRNAs, enhancing autophagy flux[46]. To explore FTO's role in our pathway, we compared RNA-seq data from primary neurons in control, injury, and injury plus m6A inhibitor (Stm2457) treatment groups[47], [48]. The results indicated reduced Actl6a expression post-injury, which was reversed by Stm2457 treatment, suggesting m6A methylation plays a key role in regulating Actl6a mRNA stability (Fig. 7A).

Dot blot experiments revealed increased m6A levels after injury (Fig. 7D-G). Altering Fto expression in cell models demonstrated that Fto overexpression decreased m6A levels, while Fto KD increased them (Fig. 7F-K), indicating Fto's regulatory role in m6A modifications.

RNA immunoprecipitation (RIP) assays confirmed a direct interaction between Fto and Actl6a mRNA, with Fto overexpression enhancing this binding (Fig. 7L, M). Methylated RNA immunoprecipitation (meRIP)-qPCR showed increased m6A modifications on Actl6a mRNA following Fto KD, supporting Fto's role in m6A demethylation (Fig. 7N, O).

Western blot and mRNA stability assays revealed that Fto KD decreased Actl6a expression and mRNA stability (Fig. 7P-R). Collectively, these results demonstrate that Fto directly modulates Actl6a mRNA expression and stability via m6A demethylation, underscoring the importance of m6A modification in neural responses to injury.

## Discussion

This study explored the role of Actl6a in spinal cord injury (SCI), focusing on how it promotes cell survival by regulating autophagy. Through in vitro experiments and cellular analyses, we confirmed that the interaction between Actl6a and Sox2 regulates the expression of key autophagy genes, Atg5 and Atg7. This regulation is crucial for the survival of damaged neural cells. Additionally, we uncovered new insights into how Fto modulates Actl6a mRNA stability via m6A methylation mechanisms.

Actl6a is a key component of the SWI/SNF complex, which is essential for chromatin remodeling and gene expression involved in development and cell fate decisions[16], [22], [49] Our study advances the understanding of Actl6a beyond its developmental roles, demonstrating its importance in neural precursor differentiation and the survival of mature neural cells. Specifically, in SCI, Actl6a is involved in recovery processes, offering potential therapeutic avenues. Autophagy is essential in SCI for maintaining cellular homeostasis by degrading and recycling cellular debris and protein aggregates[50], [51], [52]. Our experiments demonstrated that Actl6a, in conjunction with Sox2, plays a significant role in this process by regulating the expression of autophagy-related genes Atg5 and Atg7. Through CHIP-qPCR and dual-luciferase assays, we established that Actl6a and Sox2 not only co-regulate these genes but also enhance autophagy, reducing cell death after injury.

Our research underscores the critical role of autophagy in regulating cell survival after SCI. Autophagy and apoptosis are interconnected processes: autophagy protects cells by removing damaged components, while apoptosis occurs when cells cannot repair damage. Our findings suggest that Actl6a and Sox2 not only regulate autophagy but also balance autophagy and apoptosis. Overexpressing Actl6a and Sox2 reduced apoptotic markers (cleaved-Caspase-3 and Bax/Bcl-2 ratio), indicating that enhanced autophagy can protect cells from apoptosis. However, excessive or dysregulated autophagy may lead to autophagic cell death.

This synergy between Actl6a and Sox2 operates through their joint action on the promoter regions of autophagy-related genes, such as Atg5 and Atg7, which are essential components in the formation of autophagosomes. By finely tuning the transcriptional activity of these genes, Actl6a and Sox2 ensure the proper formation and maturation of autophagosomes, a key step in the autophagy process.

By promoting autophagic flux and preventing excessive apoptosis, Actl6a and Sox2 help maintain a critical balance that is necessary for tissue repair and regeneration after SCI. Atg5 and Atg7, through their roles in autophagosome formation, directly influence the capacity of cells to remove damaged organelles, misfolded proteins, and other harmful substances that accumulate during SCI. This efficient clearance is essential for preventing cellular stress and apoptosis. Understanding the balance between autophagy and apoptosis is crucial for developing therapeutic strategies aimed at improving recovery, as targeting Actl6a and Sox2 could better manage autophagy activity at different stages of SCI recovery, thereby enhancing tissue repair and functional recovery.

The interaction between Actl6a and Sox2 is key to regulating autophagy by controlling the expression of core autophagy proteins Atg5 and Atg7. This finding is consistent with earlier research showing Sox2′s role in inducing autophagy, such as upregulating Atg10 to promote cellular senescence and reduce malignancy in cancer cells[53]. Similarly, studies have shown that Sox2 regulates autophagy pathways in colorectal and bladder cancers, promoting chemotherapy resistance and cancer stem cell traits[54], [55]. Moreover, Actl6a has been implicated in autophagy and immune responses, such as in major depressive disorder, where it affects mitochondrial autophagy and immune infiltration[56].

Methylation plays a pivotal role in SCI, regulating gene expression and influencing cellular survival strategies, including autophagy, inflammatory responses, and neural regeneration[57]. Methylation regulation can optimize neural cell repair and functional recovery, which is critical for post-injury recovery[58], [59], [60]. Fto contributes significantly to these methylation dynamics. Research has shown that in models of neurodegenerative diseases, FTO affects the survival of neurons by regulating the m6A status of Nrf2[61]. Furthermore, observations from stroke models indicate that activating Fto to reduce m6A levels in the injury area can decrease cell death and promote functional recovery, further supporting the role of Fto in neural injury and repair[62]. Our study demonstrates that Fto influences Actl6a expression by modulating m6A methylation, impacting gene expression crucial for neural recovery after SCI.

The interaction of Fto with Actl6a mirrors its known effects on autophagy-related genes. Previous studies have shown that Fto demethylates Atg5 and Atg7 mRNAs, preventing YTHDF2 from binding, which enhances the transcription and translation of these mRNAs, thereby increasing autophagy flux[46]. Our research extends these findings, revealing that Fto not only affects Atg5 and Atg7 but also significantly regulates Actl6a through similar m6A-dependent mechanisms.

By investigating the Fto/Actl6a/Sox2/Atg5/Atg7 pathway, we underscore the critical role of these interactions in modulating cellular responses to SCI. Future research should explore these molecular interactions further to develop precise therapeutic strategies for SCI and other neurological conditions, providing hope for improved patient outcomes through the regulation of autophagy.

Compliance with ethics requirements

All Institutional and National Guidelines for the care and use of animals (fisheries) were followed.

## Declaration of competing interest

The authors declare that they have no known competing financial interests or personal relationships that could have appeared to influence the work reported in this paper.
