# Supplementary material for: Actl6a regulates autophagy via Sox2-dependent Atg5 and Atg7 expression to inhibit apoptosis in spinal cord injury
Source: J Adv Res. 2025 Jan 26;77:281–96. doi: 10.1016/j.jare.2025.01.038 (PMC12627868; doi:10.1016/j.jare.2025.01.038)
Supplement: Supplementary Data 1 [file mmc1.docx]

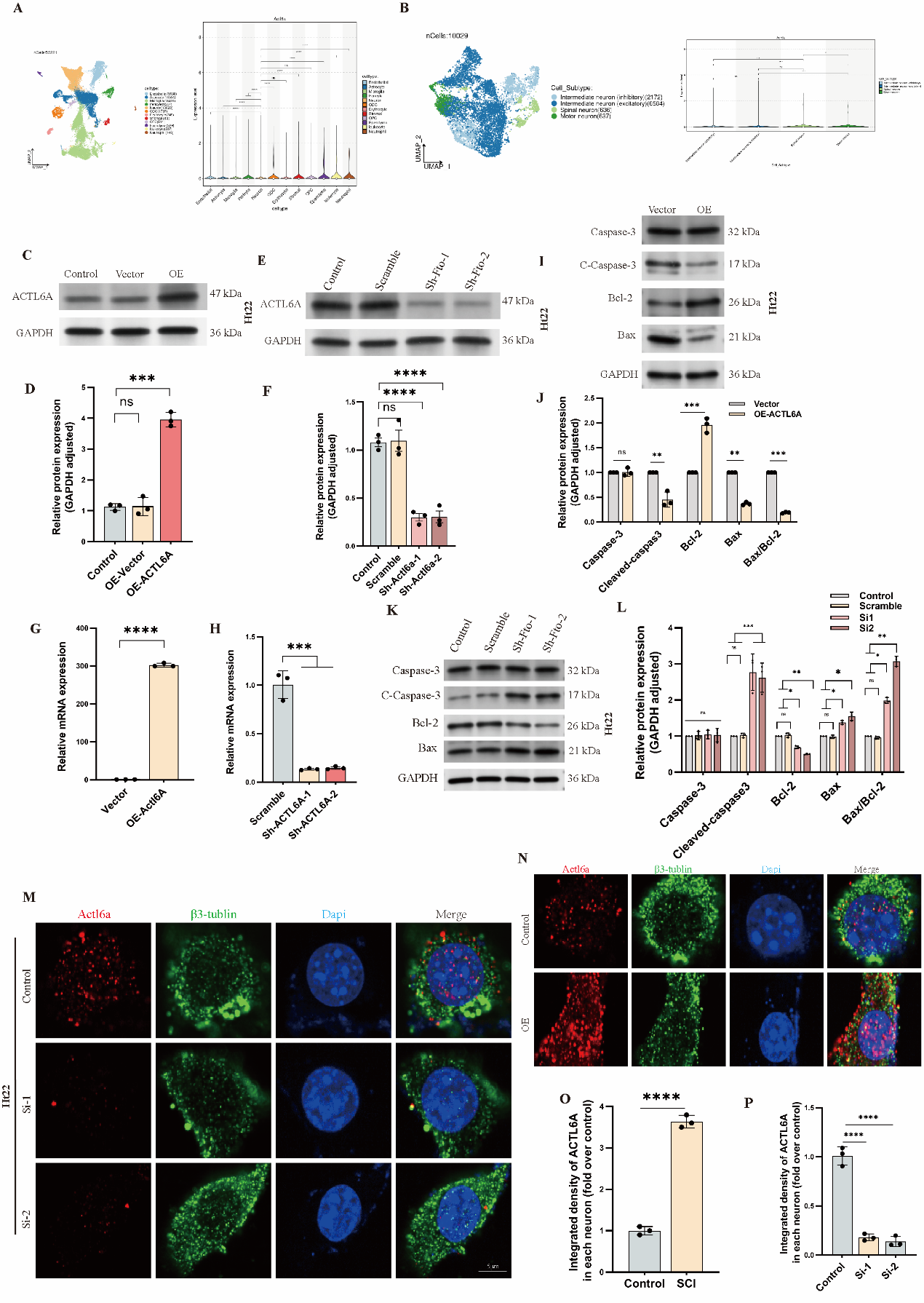


Figure S1: Regulation of Actl6a Expression and Its Impact on Apoptotic Protein Levels in HT22 Cells. (A) Single-cell clustering analysis, showing classification into neurons , oligodendrocytes (ODC), and oligodendrocyte precursor cells (OPC), while the supporting cell group includes astrocytes, microglia, endothelial cells, pericytes , stromal cells, and ependymal cells, the expression of Actl6a across these cell types. (B)Single-cell clustering analysis, showing classification into motor neurons, excitatory interneurons, inhibitory interneurons, and other spinal cord neurons, the expression of Actl6a across these cell types. (C-D) Western blot analysis of Actl6a protein levels post-overexpression in HT22 cells, with densitometric quantification shown in (D). (E-F) Western blot analysis of Actl6a protein levels post-knockdown in HT22 cells, with quantification in (F). (G-H) qPCR analysis of Actl6a mRNA levels post-overexpression (G) and knockdown (H) in HT22 cells. (I-J) Western blot analysis of apoptotic proteins (caspase-3, cleaved-caspase-3, Bax, Bcl-2) following Actl6a overexpression in HT22 cells, with quantification in (J). (K-L) Western blot analysis of apoptotic protein levels post-Actl6a knockdown in HT22 cells, with quantification in (L). (M-N) Immunofluorescence (IF) analysis of Actl6a expression post-overexpression in HT22 cells (M), with fluorescence quantification shown in (N). (O-P) IF analysis of Actl6a expression following knockdown in HT22 cells (O), with quantification in (P). The data are presented as the means ± SDs (n = 3 per group); *P < 0.05, **P < 0.01, and ***P < 0.001 indicate significant differences; ns, not significant. Significance was calculated using two-way ANOVA followed by Tukey's multiple comparison test or independent samples t-test.


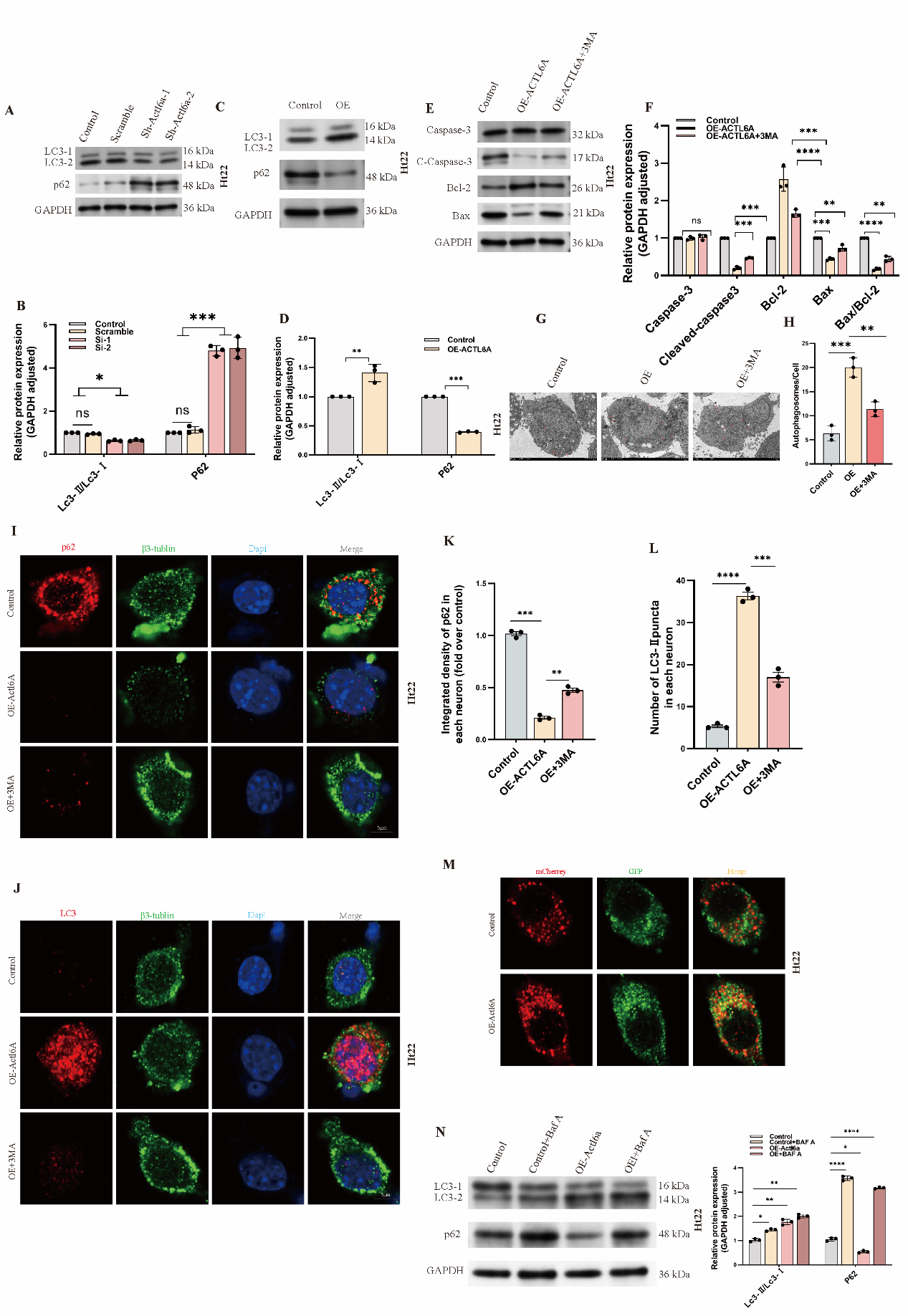


Figure S2: Analysis of Autophagy and Apoptosis Regulation by Actl6a. (A-B) Western blot analysis of autophagy-related proteins (LC3-I, LC3-II, p62) post-Actl6a knockdown in HT22 cells, with quantification in(B).(C-D) Western blot analysis of autophagy-related proteins post-Actl6a overexpression in HT22 cells, with quantification in (D). (E-F) Western blot of apoptotic proteins in HT22 cells under control, OE, and OE+3MA conditions, with quantification in (F). (G-H) Transmission electron microscopy (TEM) visualization of autophagosomes in HT22 cells, with quantification in (H). (I-J) IF analysis of p62 in HT22 cells under control, OE, and OE+3MA conditions, scale bar: 5 μm, with quantification in (J). (K-L) IF analysis of LC3 expression in HT22 cells under control, OE-Actl6a, OE-Actl6a+3MA, scale bar: 5 μm, with quantification in (L). (P-R) IF analysis of LC3 expression in primary neurons under control, OE, OE+3MA, with quantification in (R). (M) HT22 cells were transfected with pCMV-mCherry-GFP-LC3B plasmid to monitor autophagic flux. Enhanced yellow fluorescence in the OE-Actl6a group indicates increased autophagosome-lysosome fusion. (N) Western blot analysis of LC3-II and p62 in four groups: Control, Control + Baf, OE-Actl6a, and OE-Actl6a + Baf. Bafilomycin A treatment significantly increased LC3-II levels in the OE-Actl6a + Baf group, suggesting enhanced autophagic flux. The data are presented as the means ± SDs (n = 3 per group); *P < 0.05, **P < 0.01, and ***P < 0.001 indicate significant differences; ns, not significant. Significance was calculated using two-way ANOVA followed by Tukey's multiple comparison test or independent samples t-test.


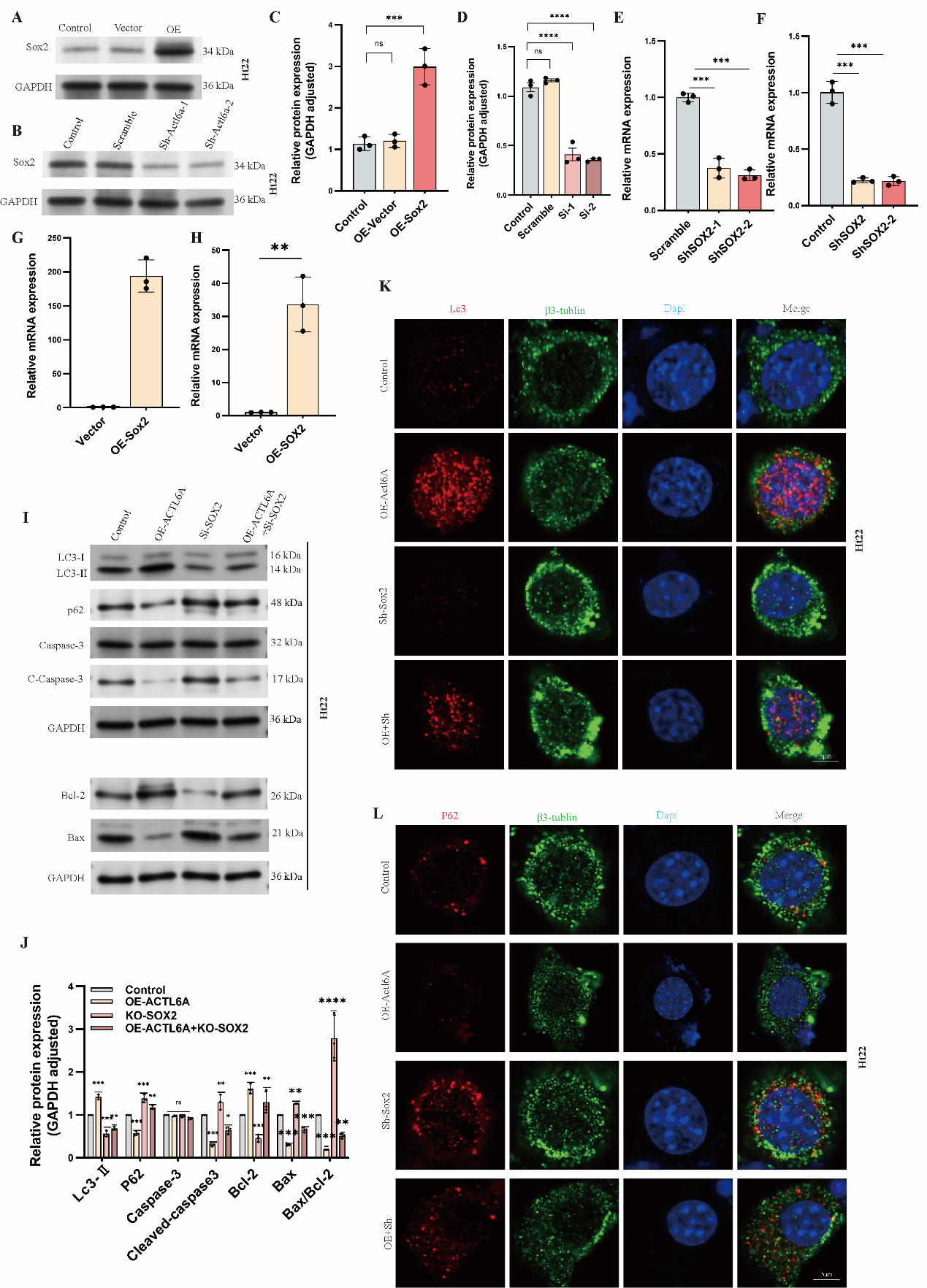


Figure S3: SOX2 Modulation and Its Impact on Autophagy and Apoptosis.
(A, C) Western blot analysis of SOX2 protein levels post-overexpression in HT22 cells, with quantification in (C). (B, D) Western blot analysis of SOX2 protein levels post-knockdown in HT22 cells, with quantification in (D). (E-H) qPCR analysis of SOX2 mRNA levels post-knockdown and overexpression in HT22 cells and primary neurons. (I-J) Western blot analysis of autophagy and apoptosis-related proteins in HT22 cells under OE-Actl6a, Sh-Sox2, and combined conditions, with quantification in (J). (K-L) IF analysis of LC3 and p62 expression in HT22 cells under OE-Actl6a, Sh-Sox2, and combined conditions, scale bar: 5 μm. The data are presented as the means ± SDs (n = 3 per group); *P < 0.05, **P < 0.01, and ***P < 0.001 indicate significant differences; ns, not significant. Significance was calculated using two-way ANOVA followed by Tukey's multiple comparison test or independent samples t-test.


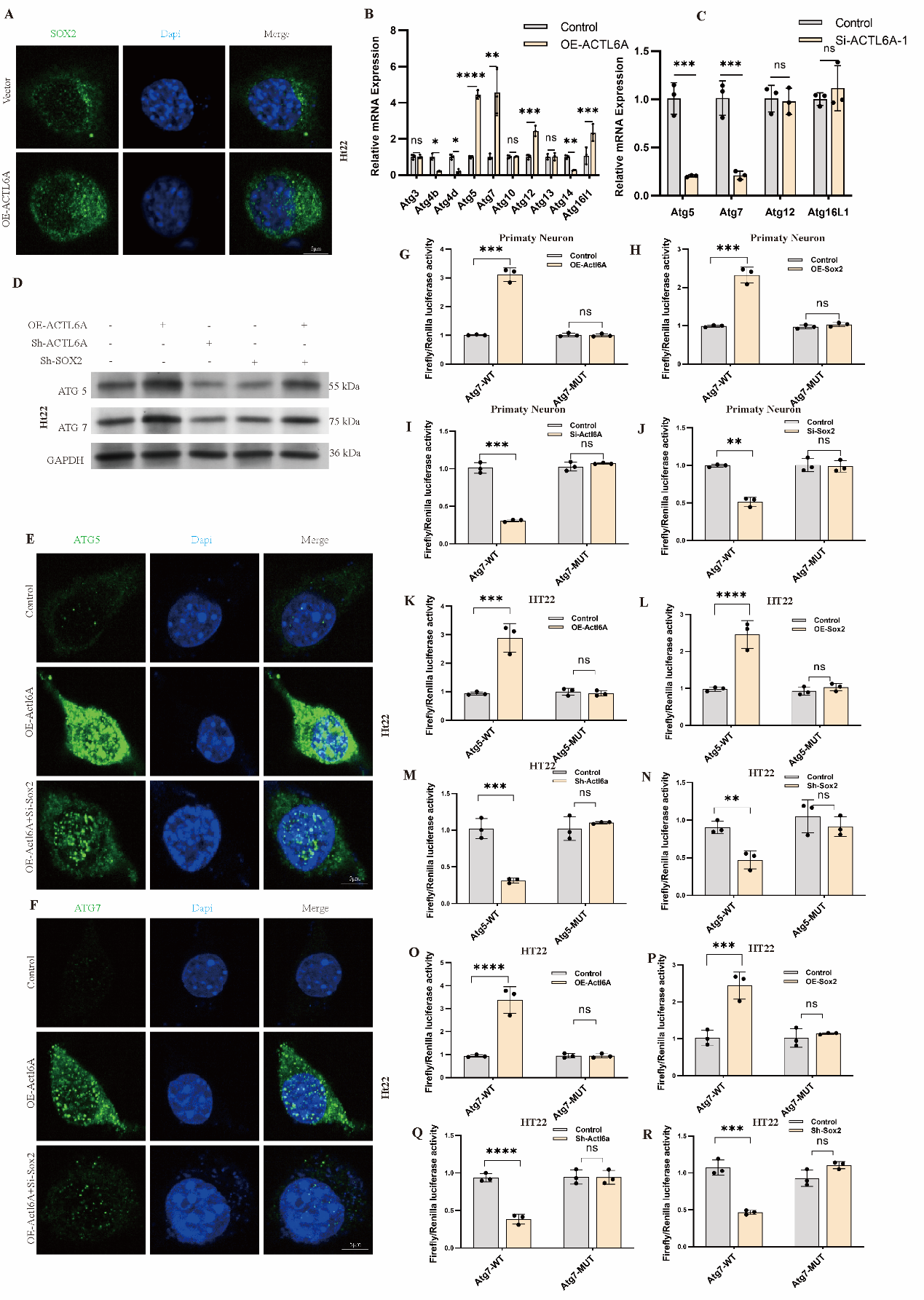


Figure S4: Actl6a and Sox2 Modulation and Its Impact on Autophagy-Related Genes.
(A) IF analysis showing co-localization of Actl6a and Sox2 in primary neurons and HT22 cells, scale bar: 5 μm. (B-C) qPCR analysis of autophagy-related gene expression post-Actl6a overexpression and knockdown in HT22 cells. (D) Western blot analysis of Atg5 and Atg7 protein levels post-Actl6a and Sox2 modulation. (E-F) IF analysis of Atg5 and Atg7 in HT22 cells post-Actl6a overexpression and Sox2 knockdown, scale bar: 5 μm. (G-N) Relative luciferase activity of Atg7 and Atg5 promoters (Wt and Mut) following Actl6a and Sox2 overexpression or knockdown in HT22 cells. The data are presented as the means ± SDs (n = 3 per group); *P < 0.05, **P < 0.01, and ***P < 0.001 indicate significant differences; ns, not significant. Significance was calculated using two-way ANOVA followed by Tukey's multiple comparison test or independent samples t-test.


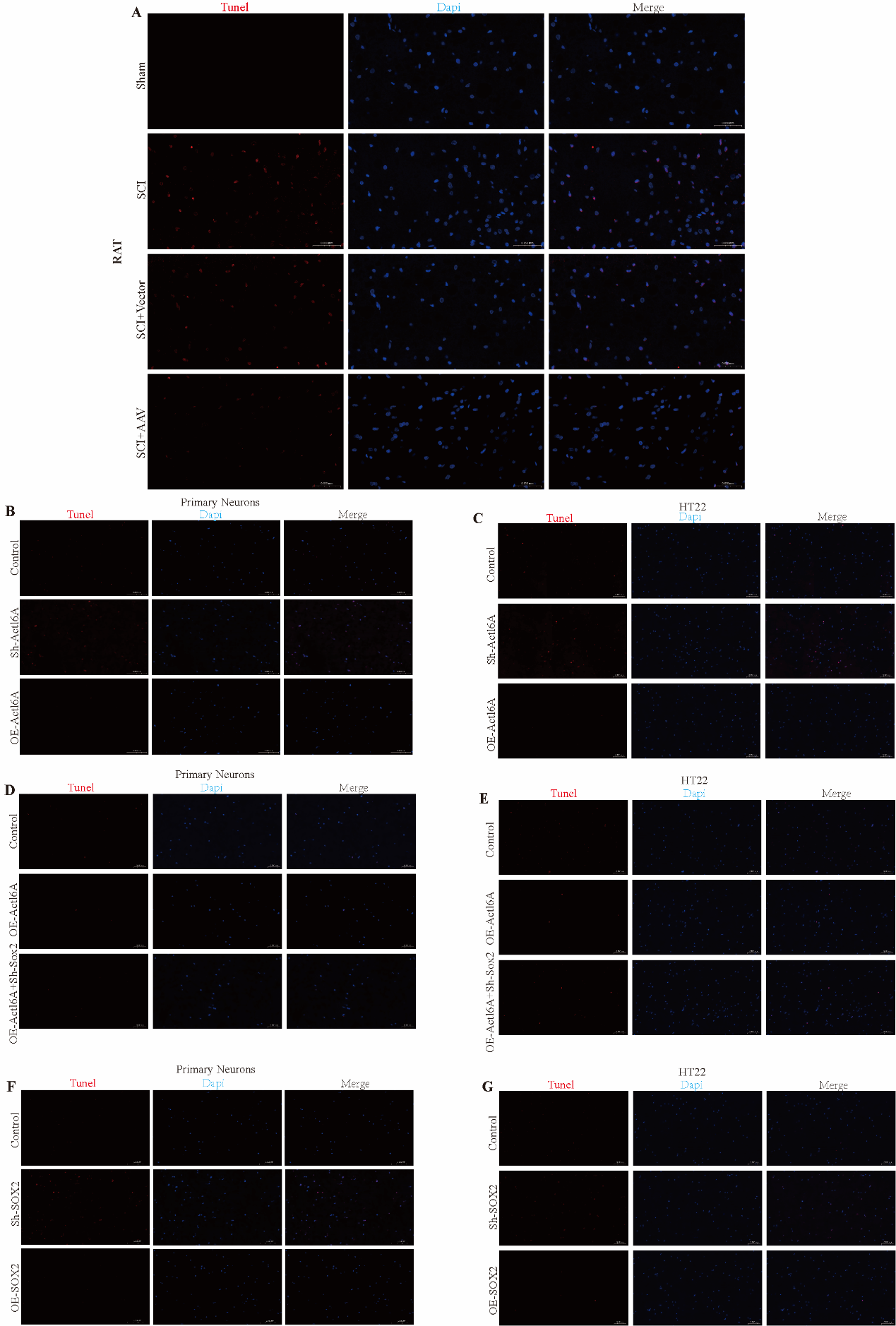


Figure S5: TUNEL Staining in Response to Actl6a and SOX2 Modulation.
(A-C) TUNEL staining of rat spinal cord, scale bar: 50 μm; primary neurons, scale bar: 200 μm; and HT22 cells, scale bar: 100 μm; post-Actl6a modulation. (D-G) TUNEL staining of primary neurons, scale bar: 200 μm; and HT22 cells, scale bar: 100 μm post-Actl6a and Sox2 modulation.


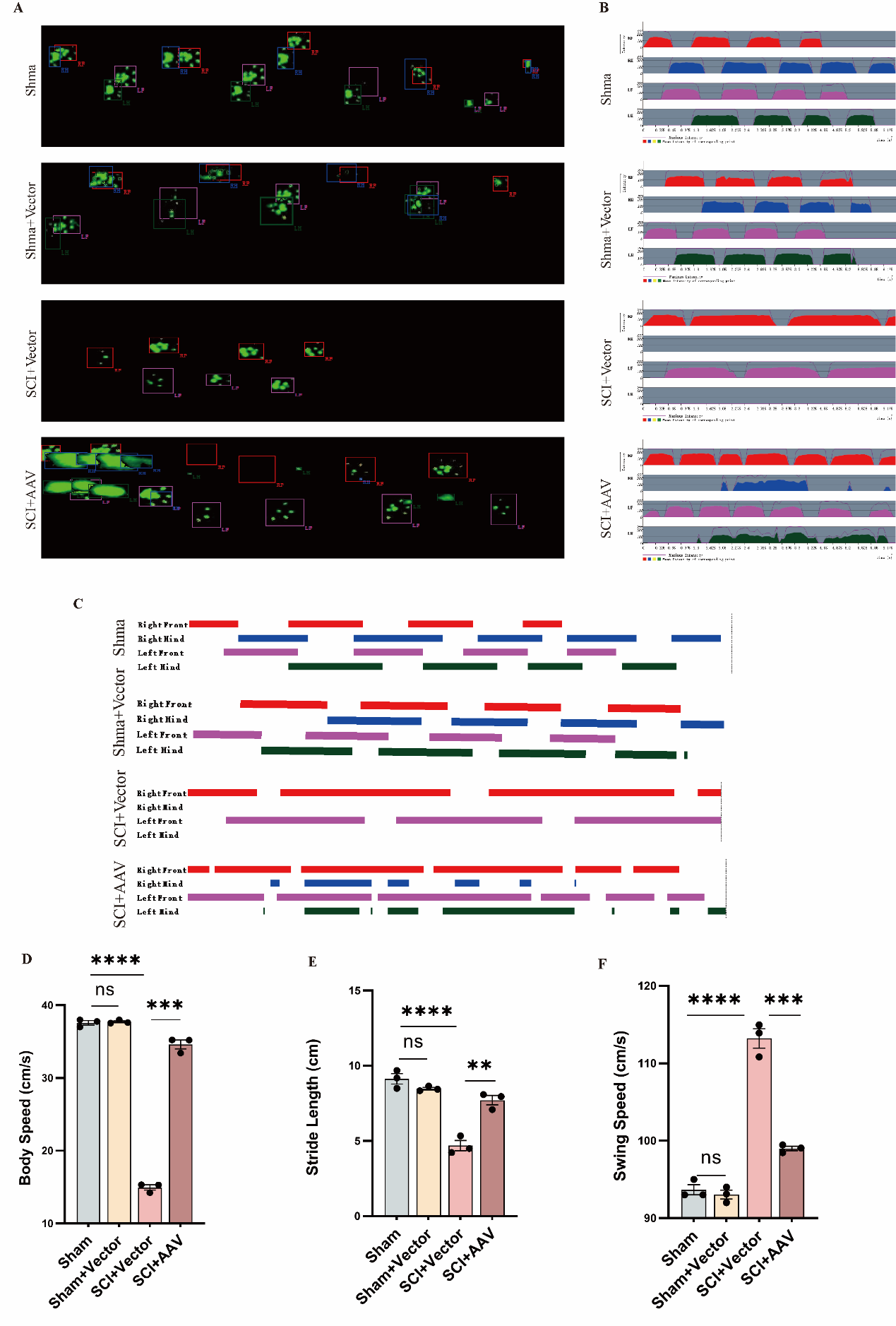


Figure S6: Gait Analysis in Mice 28 Days Post-Spinal Cord Injury. (A) Representative footprint images used for gait analysis. (B) Print Intensity analysis of paw pressure during locomotion.
(C) TimingView graphical representation of paw contact sequence during walking. (D) Body Speed analysis measuring locomotor activity. (E) Stride Length analysis reflecting gait stability.
(F) Swing Speed analysis of paw movement during the swing phase.


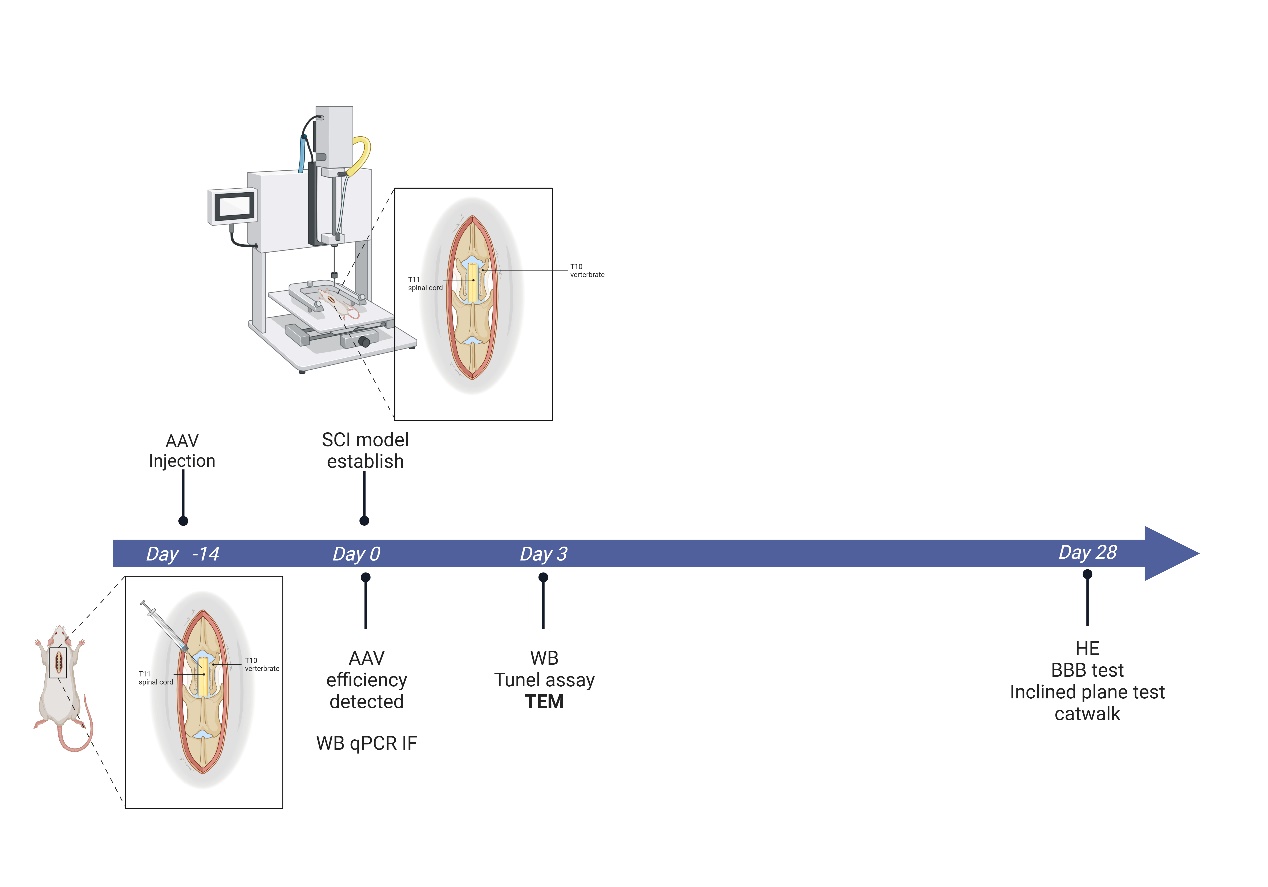


Figure S7: Experimental scheme of Rats.

**Table S1.** Sequences of qPCR primers.

| **Gene** | **Species** | **Forward primer (5’-3’)** | **Reverse primer (5’-3’)** |
| --- | --- | --- | --- |
| *Fto* | mouse | GAGCAGCCTACAACGTGACT | GAAGCTGGACTCGTCCTCAC |
| *Atg3* | mouse | GGCAGTTTTTGACTCCCCTG | TTCCCCTGTAGCCCTCTTCT |
| *Atg4b* | mouse | GATGGACGCAGCCACTTT | TCTGCCCAGAATCCAAACAG |
| *Atg4d* | mouse | CCCCGGCATTCACTGTACTT | TGGCAAAGGCCATCTCCAG |
| *Atg5* | mouse | TGTGCTTCGAGATGTGTGGTT | GTCAAATAGCTGACTCTTGGCAA |
| *Atg5(chip)* | mouse | TTATGAAGGCCAACCCAGCT | CCCATCCTCCGATCTGACCT |
| *Atg7* | mouse | GCTGCTGAGATCTGGGACAT | GAGATGTGGAGATCAGGACCAG |
| *Atg7(chip)* | mouse | ACTAGCTCCCATTTGCCTTT | TGCCCCAAACATCACAGAGA |
| *Atg10* | mouse | AGGTCAGGGCGAGCGA | CCATCGCCTATCTGCTGTGA |
| *Atg12* | mouse | TGAATCAGTCCTTTGCCCCT | CATGCCTGGGATTTGCAGT |
| *Atg13* | mouse | TCCTATCAGCCTGCTGTCCT | CTGGTGAGCGTGTGTAGTGT |
| *Atg14* | mouse | TTACAGACTGAGTGCGCTGG | CCGTGCTGGTCACTTACTGT |
| *Atg16l1* | mouse | TCCGAATCTCCCCTTTTGGGA | CTGCACTGCGTTGACCTCTC |
| *Gapdh* | mouse | GACTCCACTCACGGCAAATTCAAC | GACACCAGTAGACTCCACGACATAC |
| *Actl6a* | mouse | ATGTGTGATATTGACATCAGACCAG | CGCCAATCCATGAGCTAAACC |
| *Sox2* | mouse | CGGCGGCAACCAGAAGAACAG | CGCTTGCTGATCTCCGAGTTGTG |
|  |  |  |  |
|  |  |  |  |
|  |  |  |  |

**Table S2** Antibody.

| ANTIBODY NAME | **BRAND** |
| --- | --- |
| *Actl6a* | *Abcam，ab3882* |
| *Sox2* | *Abcam，ab171380* |
| *Fto* | *Abcam，ab280081* |
| *Caspase-3* | *Abcam, ab32351* |
| *Cleaved-Caspase-3* | *Abcam, ab2302* |
| *Bax* | *Abcam, ab32503* |
| *Bcl-2* | *Abcam, ab182858* |
| *Lc3* | *CST, Cat# 13082* |
| *P62* | *Affinity, Cat# AF5384* |
| *Atg5* | *Abcam, ab108327* |
| *Atg7* | *Abcam, ab133528* |
| *IgG*  *M6A*  *Gapdh* | *Abcam, ab172730*  *Synaptic Systems, 202003*  *Abcam, ab8245* |
|  |  |
|  |  |
|  |  |
|  |  |
|  |  |
|  |  |
|  |  |
|  |  |
