# Supplementary material for: Actl6a regulates autophagy via Sox2-dependent Atg5 and Atg7 expression to inhibit apoptosis in spinal cord injury
Source: J Adv Res. 2025 Jan 26;77:281–96. doi: 10.1016/j.jare.2025.01.038 (PMC12627868; doi:10.1016/j.jare.2025.01.038)
Supplement: Supplementary Data 2 [file mmc2.pdf]

细胞遗传质量鉴定检测

Cell Line Authentication Service

STR 基因型检测报告

检品名称：细胞系

委托单位：上海翼和应用生物技术有限公司

报告日期：2022/06/16

上海翼和应用生物技术有限公司联系地址：上海市松江区龙腾路 1015 弄中星创意园 2 号 502 室
网址：http://www.biowing.com.cn/邮箱：market@biowing.com.cn 服务热线：021-33559491

报 告 说 明

1. 本报告只对送检的来样负责。
2. 检验报告上的检验结果和检验单位名称，未经同意不得用于广告、评优及商业宣传。
3. 对本报告有异议，请于收到报告之日起十五日内以书面方式提出，逾期不予受理。
4. 对纸质检验报告涂改、增删，或未加盖检验单位印章的复印件均无效。

样 品 信 息

样品编号：

客户样本编号	公司编号
HT-22 220407E63	20220613-04

样品数量：1

样品性状：细胞系

检测项目：STR

检测方法：用 Axygen 的基因组抽提试剂盒提取 DNA，采用 10- STR 扩增方案扩增，在 ABI 3730XL 型遗传分析仪上对 STR 位点和性别基因 Amelogenin 进行检测。

上海翼和应用生物技术有限公司联系地址：上海市松江区龙腾路 1015 弄中星创意园 2 号 502 室
网址：http://www.biowing.com.cn/邮箱：market@biowing.com.cn 服务热线：021-33559491

检 测 结 果

(一) 检验基本情况

编号	多等位基因	匹配细胞系	人源污染	与对比细胞匹配度 EV 值	匹配说明
20220613-04	有	HT22	否	1.0	完全匹配

样本基因型检验结果

- 多等位基因指三等位及以上基因现象。
- 本次检测各细胞分型结果良好。

(二) 各样本描述

- 20220613-04：该株细胞鉴定结果为**小鼠细胞系**，EXPASY 数据库显示和细胞系 **HT22 完全匹配**，细胞号 **CVCL_0321**。本次检测在该细胞系中，**发现多等位基因**。（因数据库未登录小鼠细胞系相关 STR 数据信息，数据库匹配的是 HT22）

备注：待测细胞系与收录于 ATCC, DSMZ, JCRB 和 RIKEN 数据库的细胞系 STR 数据进行比对，未收录于以上细胞库的细胞系将无法匹配。下列位点中 D4S2408 为人源位点，用于检测该细胞是否有人源污染。

上海翼和应用生物技术有限公司联系地址：上海市松江区龙腾路 1015 弄中星创意园 2 号 502 室
网址：http://www.biowing.com.cn/邮箱：market@biowing.com.cn 服务热线：021-33559491

（三）样本分型结果

细胞 20220613-04 的 STR 位点和 Amelogenin 位点的基因分型结果

Loci	送检细胞 STR 信息				细胞库细胞 STR 信息		
	送检细胞名：HT-22 220407E63				细胞库细胞名：HT22		
	Allele1	Allele2	Allele3	Allele4	Allele1	Allele2	Allele3
4-2	229.91 [18.3]	233.97 [19.3]	238.02 [20.3]		18.3	19.3	20.3
5-5	332.14 [13]	336.2 [14]	340.17 [15]		13	14	15
6-4	291 [15.3]				15.3		
6-7	334.75 [12]				12		
9-2	221.53 [15]	225.6 [16]					
12-1	242.53 [20]				20		
15-3	193.27 [20.3]				20.3		
18-3	156.57 [17]	164.72 [19]			17	19	
X-1	400.39 [25]				25		
D4S2408							

上海翼和应用生物技术有限公司联系地址：上海市松江区龙腾路 1015 弄中星创意园 2 号 502 室
网址：http://www.biowing.com.cn/邮箱：market@biowing.com.cn 服务热线：021-33559491

其他说明

(一)　分型方案及位点分布

	方案 1	方案 2
1	18-3(FAM)	12-1(FAM)
2	4-2 (FAM)	5-5(FAM)
3	6-7(FAM)	X-1(FAM)
4	9-2(NED)	15-3(NED)
5		6-4(NED)
6		D4S2408(NED)

实验方案及位点

主要实验人员：张佳男

复核人: 张晨茜

负责人：巢凯悦

签发日期：2022 年 06 月 16 日

上海翼和应用生物技术有限公司联系地址：上海市松江区龙腾路 1015 弄中星创意园 2 号 502 室
网址：http://www.biowing.com.cn/邮箱：market@biowing.com.cn　　服务热线：021-33559491

附件一：HT-22 220407E63 数据库比对

Accession	Name	№ Markers	Score	STR 1-1	STR 1-2	STR 2-1	STR 3-2	STR 4-2	STR 5-5	STR 6-4	STR 6-7	STR 7-1	STR 8-1	STR 11-2	STR 12-1	STR 13-1	STR 15-3	STR 17-2	STR 18-3	STR 19-2	STR X-1
**NA**	**Query**	**NA**	**NA**					**18.3,19.3,20.3**	**13,14,15**	**15.3**	**12**				**20**		**20.3**		**17,19**		**25**
CVCL_0321	HT22	8	100.00%	10,11	13,17	9	14,15	18.3,19.3,20.3	13,14,15	15.3	12	29	15	15,17,18	20	16.2	20.3	12,13,14	17,19	11,12	25

上海翼和应用生物技术有限公司联系地址：上海市松江区龙腾路 1015 弄中星创意园 2 号 502 室
网址：http://www.biowing.com.cn/邮箱：market@biowing.com.cn 服务热线：021-33559491

附件二：HT-22 220407E63 测序峰图

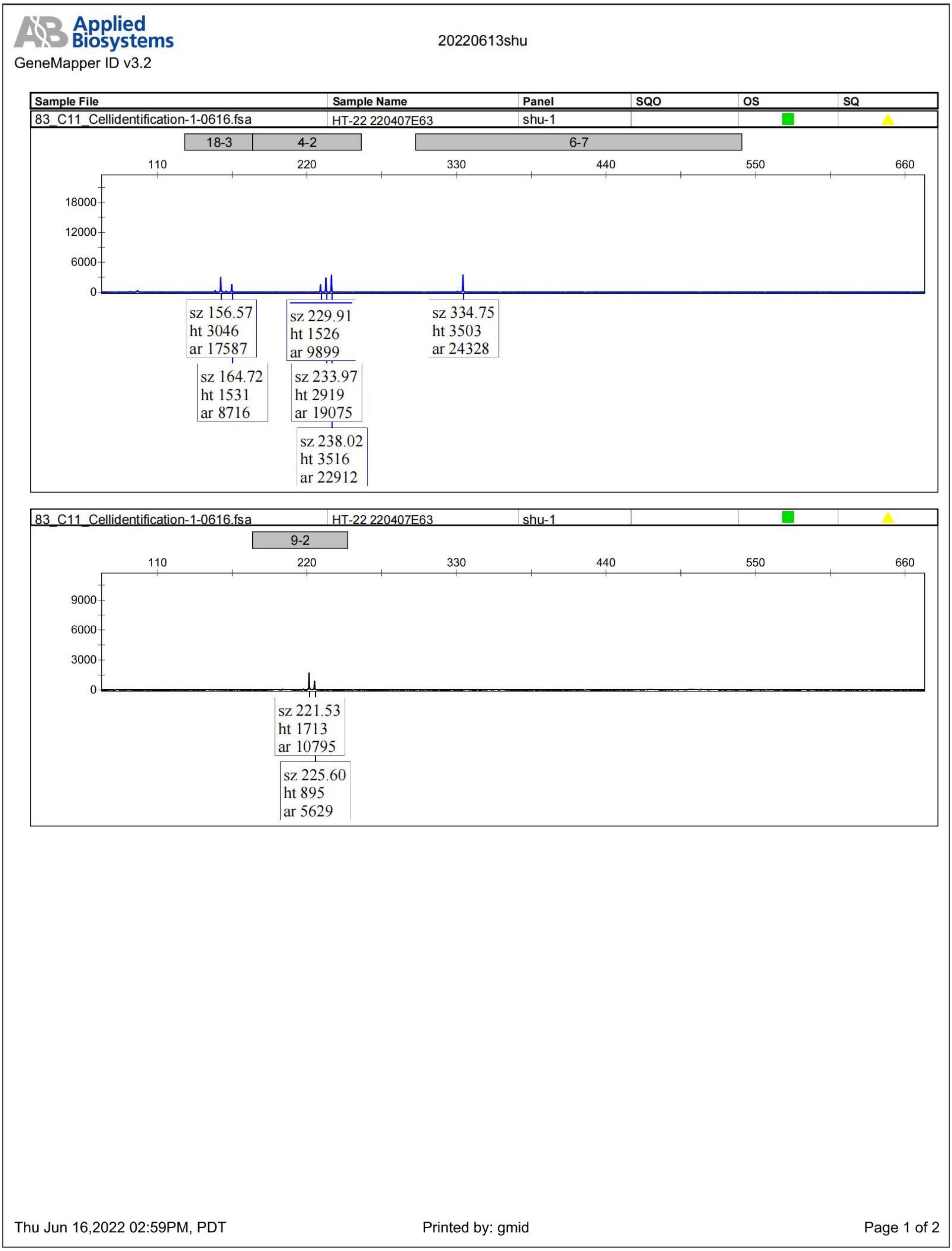

Applied Biosystems
GeneMapper ID v3.2

20220613shu

Sample File	Sample Name	Panel	SQO	OS	SQ
91_C12_Cellidentification-1-0616.fsa	HT-22 220407E63	shu-2			

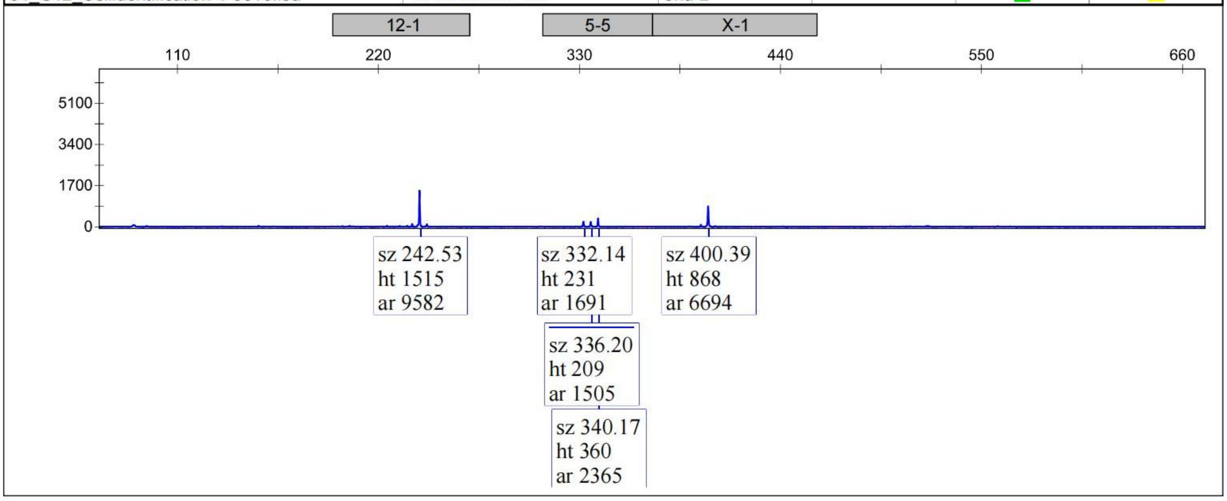

91_C12_Cellidentification-1-0616.fsa	HT-22 220407E63	shu-2			

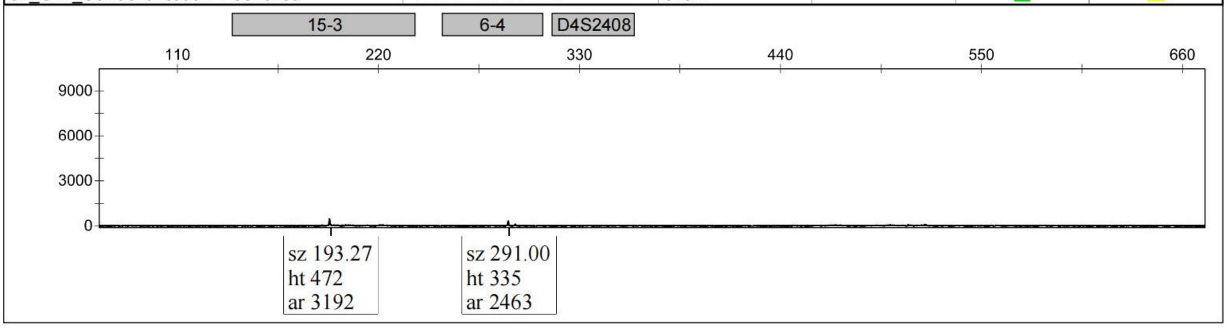

Thu Jun 16,2022 02:59PM, PDT Printed by: gmid Page 2 of 2
